# Determinants of dairy products purchase decisions among polish doctors: A gender-based analysis

**DOI:** 10.1371/journal.pone.0339849

**Published:** 2026-02-27

**Authors:** Anna Nowak, Aneta Jarosz-Angowska, Artur Krukowski, Agnieszka Komor, Anna Goliszek, Sebastian Białoskurski, Bartosz G. Sołowiej, Katarzyna E. Przybyłowicz, Katarzyna Staniewska, Aneta Dąbrowska

**Affiliations:** 1 Department of Economics and Agribusiness, Faculty of Agrobioengineering, University of Life Sciences in Lublin, Lublin, Poland; 2 Department of Management and Marketing, Faculty of Agrobioengineering, University of Life Sciences in Lublin, Lublin, Poland; 3 Department of Dairy Technology and Functional Foods, Faculty of Food Sciences and Biotechnology, University of Life Sciences in Lublin, Lublin, Poland; 4 Department of Human Nutrition, Faculty of Food Sciences, University of Warmia and Mazury in Olsztyn, Olsztyn, Poland; 5 Department of Commodity Science and Food Analysis, Faculty of Food Science, University of Warmia and Mazury in Olsztyn, Olsztyn, Poland; 6 Department of Dairy Science and Quality Management, Faculty of Food Science, University of Warmia and Mazury in Olsztyn, Olsztyn, Poland; Lusofona University of Humanities and Technologies: Universidade Lusofona de Humanidades e Tecnologias, PORTUGAL

## Abstract

The study aimed to address a research gap by identifying the factors that influence the purchasing decisions of individual Polish doctors in the dairy products market, with gender as a demographic variable. The primary data collection method was a survey, conducted using the CAPI (Computer-Assisted Personal Interviewing) technique. The sample was selected using a purposive approach with elements of proportional territorial allocation. The survey involved 201 interviews with doctors from across Poland representing various fields of medical specialisation. The group of respondents surveyed is characterised by one of the highest levels of social trust. Conducting research among a professional group of doctors who are perceived as experts, whose knowledge is based on scientific evidence and many years of education, is an important contribution to the discussion on the factors that shape purchasing behaviour. The research confirmed the hypothesis that purchasing decisions in the dairy market are shaped by diverse factors. The study showed that the surveyed group of doctors most often makes purchasing decisions guided not only by health and quality values, but also by pragmatic ones, such as a product’s shelf life or availability in shops. The conducted research showed that differences between women and men representing the medical community in the assessment of factors influencing purchasing decisions in the dairy products market occur for 6 of the 26 variables assessed, thus one of the research hypotheses was only partially confirmed. Further research using principal component factor analysis made it possible to determine what hidden factors influence purchasing decisions among all respondents and groups distinguished by the demographic characteristic of gender, which allowed for the definition of customer segments.

## 1. Introduction

The dairy market is one of the fastest-growing food markets. Milk and dairy products play an important role in the human diet and form part of official dietary recommendations in many countries worldwide [1–3]. The global dairy market reached a value of USD 991.5 billion in 2024, and forecasts indicate further growth of 4.75% per annum on average between 2025 and 2033. This is due to the growth in the world’s population and the increasing demand for essential nutrients such as calcium and protein, which are found in large quantities in dairy products [4]. According to data from the International Dairy Federation (IDF), per capita consumption (in dairy product equivalent) globally was approximately 119.1 kg per year [5]. However, the consumption of raw milk and its products varies significantly between countries, due to different income levels and regional preferences [6]. In the European Union, raw milk production amounted to approximately 160.8 million tonnes in 2023 [7]. However, it is projected to decline as environmental regulations and low profitability are causing a reduction in dairy cattle numbers [8].

The group of milk and dairy products is notable for its important role in meeting human nutritional needs [9,10]. Consumers appreciate dairy products for their high nutritional value, health-promoting properties, and desirable sensory attributes [11,12]. They are also closely linked to the concept of sustainable consumption in the European Union, which is concerned with balancing the environmental impact of production processes with consumer demand and eating habits. Sustainable consumption involves using material goods and services to provide consumers with a better quality of life without degrading the environment or endangering the consumption of future generations. Nevertheless, the production and consumption of dairy products such as milk, cheese and butter has significant environmental implications regarding greenhouse gas emissions, land use and water consumption [13]. Within the framework of sustainable consumption, it is important to consider changes that encourage a healthy lifestyle and wholesome diet, in which dairy products play a significant role. Food policy should consider issues related to sustainable food production in the context of consumer health. Therefore, the authors’ decision to conduct research on the factors influencing the purchasing decisions of dairy product consumers on a purposively selected sample of Polish doctors and to take into account their opinion-forming role in shaping marketing strategies is of great importance and constitutes a significant contribution to scientific research.

The theoretical framework of this study is based on the Theory of Planned Behaviour (TPB), which assumes that individual behaviour is motivated by behavioural intentions shaped by attitudes, subjective norms and perceived behavioural control [14,15]. It is worth noting that this theory is used in the literature to assess the factors determining purchasing intentions and behaviours in the food market [16,17]. In the context of Polish doctors’ decisions to purchase dairy products, these components may explain how personal beliefs about health and nutrition (attitudes), social and professional expectations (subjective norms), and perceived ease or limitations in accessing specific dairy products (perceived control) influence purchasing behaviour. Gender differences may further moderate these relationships, as men and women often exhibit different patterns of health consciousness and sensitivity to social influences. The application of TPB allows for a structured interpretation of the psychological and social determinants underlying the purchasing decisions of this professional group. Consequently, this model provides a solid theoretical basis for segmenting respondents and explaining differences in consumer behaviour based on gender. The results of the study confirm that TPB is a predictive model explaining intentions and behaviours related to the purchase of dairy products [18].

Numerous studies have examined consumer behaviour in the dairy market, but the factors influencing consumer purchasing decisions are constantly changing [2,19,20]. In this context, understanding the determinants of consumer purchasing decisions, their needs and preferences, and the criteria they use to select products is crucial [2]. A review of the literature reveals that consumers’ decisions about which foods to purchase are influenced by a variety of factors. According to Grębowiec [11] the most important determinants include economic factors (income and price) and individual preferences and beliefs (taste and perceived healthiness). The socio-cultural context, including food traditions, promotional activities, and brand trust, is also significant [21]. Furthermore, studies indicate that characteristics such as age, social status and place of residence (socio-demographic factors) as well as temperament and personality (psychological factors) influence the level and type of dairy product consumption [22,23]. Some studies show that a significant part of consumer decision-making process is unconscious, influenced by individuals and entities present in the environment [24,25]. A study by Grębowiec [11] shows that consumers’ decisions to purchase dairy products are largely routine, given how frequently this type of product is bought. Conversely, there are many environmental stimuli that can positively or negatively influence dairy product consumption. Claims regarding the negative impact of cow’s milk on human health and milk production process on the environment, perceptions of poor animal welfare in milk production, as well asnew dietary styles that exclude dairy products can all negatively influence milk and dairy product consumption [26]. At the same time, there are trends towards ‘green’ consumption and the choice of traditional, local, sustainable products. These trends can therefore influence purchasing decisions relating to food products, including milk [27,28].

The ever-changing needs and preferences of consumers, the increasing range and availability of product information, the transformation of the commercial sector, and changing lifestyles all contribute to changing perceptions of, and requirements for, the food purchased [9]. The importance of milk and dairy products in human nutrition, the diversity of consumers’ attitudes, and the variety of products in this category highlight the need to explore the factors influencing the purchase of dairy products. This type of research is also important because purchase decision-making in the food market is a dynamic, complex process that evolves with changes in the socio-economic situation and cultural transformations. In the context of the aforementioned determinants influencing milk consumption, as well as the importance of dairy products in the human diet, it is particularly important to purposefully select a proper sample of respondents for study. Authors decided to target Polish doctors – consumers with a medical background who accurately know the importance of dairy products for human health. These respondents also consider many other factors when making consumer decisions about dairy products, not only health-related ones, which gives wider context of the research conducted. Including the gender of respondents is an additional advantage, as numerous studies have demonstrated the importance of gender in the consumer decision-making process [29–33]. Adapting the offer of dairy products to the actual expectations and needs of consumers, taking into account the opinion-forming role of doctors, is, on the one hand, the basis for the development of companies producing these products, and on the other hand, it can influence an increase in milk and dairy products consumption, which is very important from the food policy perspective.

Very few studies have examined the role of health professionals, including doctors, as reference points in consumers’ choices regarding dairy products. One such study was a US survey of 331 healthcare professionals which examined physicians’ preferences for recommending dairy versus plant-based beverages. While some respondents were open to recommending plant-based alternatives, but most of the doctors surveyed still considered dairy products to be an important source of calcium [34–36]. Utter et al. [37] emphasised the importance of the work environment and the availability of healthy products in shaping the eating habits of medical staff. Most of these studies cover broad groups of medical staff, not just doctors. Respondents representing the medical sector, due to their expertise, are also active participants and intermediaries in the consumer decision-making process. Gutkowska’s [38] research shows that in the hierarchy of sources of information about food and nutrition, high priority should be given to information provided by friends and family, as well as advice from doctors or dieticians.

Doctors, who enjoy a high level of public trust can influence patients’ purchasing behaviour by providing important information. Their consumer attitudes and preferences, including their opinions on the nutritional, ecological, and sensory value of dairy products, can influence consumers through direct recommendations and by setting an example through their personal choices and lifestyle. The authors were prompted to undertake this study by the fact that, to their knowledge, there are no existing studies in the literature on factors influencing consumer behaviour conducted on a group of doctors. Two dimensions of the gap in existing research on dairy products can therefore be identified. Firstly, the existing literature has paid very little attention to the role of doctors as opinion leaders influencing consumer attitudes towards dairy products, even though this is a group with high social authority and the potential to shape the food choices of others. Secondly, there is a lack of research that segments respondents by gender, which is important in light of evidence pointing to different consumer decision-making patterns between women and men. This study therefore responds to the need for empirical data that can serve as a basis for the development of effective marketing and communication strategies by entities in the dairy industry, taking into account the socio-professional context of opinion-forming by doctors not only in the area of public health, but also in the purchasing decision-making process. The results of the study may be universal for other European countries due to the convergence of food consumption patterns and the similarity of characteristics of this professional group of respondents. Doctors constitute a group with similar social status, level of education and health awareness in different European countries. Therefore, the results of the study are likely to be transferable to other European countries.

The aim of this study is to identify factors influencing the purchasing decisions of individual buyers representing the Polish medical community in the dairy products market, taking into account the demographic variable of gender, and to segment respondents with similar factor profiles, i.e., similar response patterns. A particular contribution of the study is the conduct of research in a professional group of respondents related to medicine – in particular doctors and dieticians – who play an important role in shaping the nutritional attitudes of society. Their nutritional recommendations, based on scientific knowledge and clinical experience, have the potential to exert a real influence on consumer decisions made by patients and their families, including those concerning the consumption of dairy products.

## 2. Methods

During the research investigation, three hypotheses established in the literature were verified.

H1: Purchasing decisions in the dairy market are shaped by diverse determinants for individual purchasers representing the medical profession, including health-oriented and quality motives (i.e., those related to food safety), as well as aspects of origin and product trust, economic and pragmatic considerations, marketing factors, and psychosocial factors.

The literature shows that health consciousness and perceived product quality are key determinants of purchasing behaviour, including in the dairy product segment. For example, research by Sajdakowska et al. [20] showed that quality (freshness, naturalness, nutritional value, health benefits) is one of the main factors influencing the choice of animal-based foods. Thapa et al. [39], in turn, drew attention in their research to the positive impact of food safety awareness on the behaviour of milk consumers. Therefore, in the case of doctors, who are usually characterised by higher health awareness and medical knowledge, it can be assumed that health and quality motives (including food safety) will have a stronger than average influence. Another important dimension is the origin of the product (e.g., national, local, traditional) and trust – both in the producer and in the production or labelling system (traceability). The literature indicates that consumers use heuristic cues (e.g., country of origin, locality, certifications) to assess the quality and safety of food products [40]. Research also shows that trust in information sources has a significant impact on purchasing attitudes towards milk [41]. With regard to the medical community, it can be assumed that the aspects of trust and origin will be significant, as these individuals may be sensitive to risks related to food safety, quality and origin of raw materials. The literature also reports that quality and safety are important, but consumers are still strongly influenced by costs and purchasing pragmatics (packaging, availability). Therefore, it is reasonable to include economic and pragmatic determinants in the hypothesis. Even a group of specialists (doctors) may be guided by calculation and convenience of purchase, in addition to health-related motives. The literature on milk consumption also emphasises that the intensity of promotion influences the choice and purchase of milk [1]. In the context of doctors, it can be considered that marketing communication aimed at the medical segment may have specific significance, e.g., emphasising functional characteristics, quality certificates, innovative ingredients. The literature on purchasing behaviour in the food market also emphasises the role of social and psychological factors, e.g., the influence of peer groups, consumption norms, and the perception of one’s own role as a health-conscious person [42,43]. For doctors, their professional identity may influence them to treat shopping not only as a way to satisfy individual needs, but also as an expression of a ‘health expert’ lifestyle. This may increase the importance of health, quality and social motives. As a result, doctors, as a specific group of consumers, may place greater importance on health and quality motives than average consumers, but their decisions will still be shaped by other groups of determinants. Hypothesis H1 assumes this multidimensionality, according to which purchasing decisions are determined not only by one factor, but by a set of factors.

H2: There are differences in the assessment of the motivators of purchasing decisions in the dairy market between female and male medical practitioners.

Empirical evidence from research on purchasing decisions indicates that there is a theoretically justified expectation of differences between female and male doctors in their assessment of the motivators behind purchasing decisions in the dairy products market. These differences result from a combination of biological, psychological, socio-cultural and economic factors that shape the perception of risk, health, quality and nutritional value of food [33,44]. The literature shows that, from a psychological and health perspective, women have a higher level of health awareness and are more committed to health-promoting behaviours [44,45]. Studies have repeatedly confirmed that women are more likely than men to pay attention to the composition, nutritional value and calorie content of products, as well as to labels providing information on food safety and origin [46,47]. Men, on the other hand, are more likely to be guided by pragmatic and sensory characteristics such as taste, satiety, availability and price [33,48]. With regard to dairy products, this means that women may more often value nutritional value, low fat content or quality certificates, while men may place greater emphasis on utility and economic value. In turn, the Theory of Planned Behaviour explains that differences in attitudes, subjective norms and perceived behavioural control can lead to different purchasing intentions and behaviours [49]. Accordingly, there are solid theoretical and empirical grounds for expecting gender differences in the assessment of determinants of purchasing decisions for dairy products, including among doctors.

H3: The structure of the latent factors influencing purchasing decisions in the dairy market differs according to the gender of the doctors surveyed, creating different buyer profiles.

Food purchasing decisions are not based on just one or two clearly observable motives, but often on a combination of many hidden (latent) variables that can be identified, among other things, through factor analysis or latent class analysis [50]. The literature on the milk and dairy products market also notes that motivational factors such as taste, price, origin, health composition, brand and convenience of purchase can be arranged in different configurations for different consumer groups [51]. Research shows that women and men differ not only in terms of the importance of individual purchasing motivators, but also in their arrangement. This means that different factors may be dominant or secondary for them, and they may also occur in different relationships with each other. For example, a study of functional milk drinks showed a different structure of the motivational model depending on gender [29]. Other studies on the milk market have shown that although many product characteristics are rated similarly by women and men (e.g., origin, brand), there are statistically significant differences in which attributes are key for each gender. For example, women are more likely to pay attention to fat content, while men are more likely to pay attention to information labels or brand [48]. It can therefore be assumed that the structure of latent motivational factors, indicating how motivators are related and how important they are, may differ between female and male doctors. Understanding these differences allows for better market segmentation and adaptation of marketing communication and dairy product offerings to gender specifics.

In order to achieve the objective of this article and verify the research hypotheses, empirical research was conducted. The primary data collection method was a survey. A research instrument in the form of an original questionnaire was prepared specifically for the purposes of the study. The questionnaire consisted of several questions, including demographic and specific questions. The questionnaire was prepared on the basis of the results of a cognitive and critical analysis of the literature [1,11,20,23,39,40,51–53], as well as data obtained through the use of the systematic consensus generation method (Delphi method – three rounds). Six experts representing various fields of knowledge connected with consumer behaviour, including agriculture, economics, management and sociology, were invited to participate in the panel. The experts were asked to identify and organise key purchasing motives relevant to the dairy products market. Each factor in the set identified through the above procedure was then assessed by the respondents in terms of its importance in making a purchasing decision on a 5-point Likert scale (scale: 1 – completely unimportant, 2 – rather not important, 3 – difficult to say, 4 – rather important, 5 – very important). The Likert scale is one of the most basic and frequently used psychometric tools in the social sciences [54].

The study was carried out using the CAPI (Computer-Assisted Personal Interviewing) technique among 201 final purchasers representing the health service in Poland, which ensured the completeness of the questionnaires and eliminated the problem of missing data. Purposive quota selection was used for sampling. Respondents were required to be practising medical professionals with direct patient contact. The research had a nationwide scope as it was conducted in all voivodeships (16 regions in Poland on NUTS 2 level). Doctors were selected in proportion to the population size of each region, meaning that an element of proportional territorial allocation was applied. The survey included all the main cities of the voivodeships, and the established quotas were supplemented with respondents from randomly selected smaller towns and cities in regions classified at the NUTS 3 level.. Although the sampling strategy was non-random, it ensured spatial diversity among respondents and increased the geographical representativeness of the survey results. In addition, the distribution of the structure in terms of age and gender was controlled to reflect the actual structure of the population of doctors in Poland according to data from the Central Statistical Office (GUS), which increases the accuracy of intergroup comparisons.

Although the sample selection was purposeful, the approximate maximum estimation error for a sample of this size (N = 201, confidence level 95%, p = 0.5) is approx. ± 6.9 p.p., which allows the results to be placed in the context of typical descriptive quantitative studies. In light of the objectives of the study, which was exploratory and descriptive rather than comparative and quantitative, this sample size allowed for reliable qualitative and quantitative conclusions to be drawn. No formal test power analysis was performed because the aim of the project was not to verify statistical hypotheses in an inferential sense (i.e., testing statistical hypotheses and generalising the results from the sample to the entire population), but to describe the attitudes and practices of doctors in relation to healthy lifestyles and nutritional recommendations. The sample size was therefore adjusted to the specific characteristics of the population and the nature of the study.

The questionnaire survey was conducted between 23 September 2024 and 25 April 2025. At the beginning of the survey, there was information about the project under which the research was being conducted and that the data were protected in accordance with the European General Data Protection Regulation (GDPR). Respondents were informed at the beginning of the questionnaire that they could withdraw from the survey at any time and that completing the questionnaire constituted consent to participate in the research project and confirmed that they had read and understood the research procedure. The respondents were doctors registered on special CAWI research panels of the company conducting the survey; however, due to GDPR, the company does not provide any information that could identify individual participants to researchers during or after data collection. This study was conducted according to the guidelines of the Declaration of Helsinki, and approved by the Bioethics Committee of the Faculty of Medical Sciences, University of Warmia and Mazury in Olsztyn, Poland in 2015, Resolution No. 9/2015.

Of the respondents representing health service, 120 were women (59.7% of respondents) and 81 were men (40.3%). The following age groups were surveyed: 25–34 years (14.9%), 35–44 years (31.3%), 45–54 years (30.3%) and 55 years or older (23.4%). The respondent doctors represented 23 different medical specialities, the most frequently indicated of which were internal medicine (internist), with 75 indications, and paediatrics, with 57 indications. Most respondents (92%) worked in urban areas, while 8% were employed in rural areas. The study was not representative, and the results can only be related to the study population. Among the respondents, 18% had short work experience (up to 10 years), 35% had medium work experience (10–20 years), and 47% had long work experience (over 20 years). They indicated medical clinics (70%), private practices (36%), teaching hospitals (15%), district hospitals (10%), provincial hospitals (5.5%) and specialist hospitals (7.5%) as their main places of professional practice.

A limitation of this study is its focus on a narrow group of respondents representing the Polish healthcare sector, which, on the one hand, demonstrates the originality of the study, but on the other hand, limits the possibility of generalising the results to a wider population. The study used a non-random sample selection, which did not allow for the construction of a representative sample for either the population of doctors in Poland or the general population of consumers in Poland. However, it is worth noting that the study has a number of strengths that significantly enhance its scientific and practical value. Particularly noteworthy is the purposive selection of the sample, which includes representatives of the medical community – a professional group with above-average expertise in health and nutrition. The perspective of this group can be an important reference point for analysing consumer attitudes and behaviours in the wider population, as well as a useful source of information for developing information and education policy tools and marketing communication in the dairy products market. In addition, the quota selection of respondents in proportion to the population in each province ensured regional balance and increased spatial representativeness. An additional research limitation in this study is the lack of pilot studies.

The article’s themes include the independent variable (gender of the respondents) and the dependent variable (motivators of product purchase decisions in the dairy market). As part of the survey, respondents were asked to indicate their gender (single choice) and to evaluate factors influencing dairy product purchases. To assess these factors, respondents were presented with a set of 26 variables identified through a cognitive-critical analysis of the literature [11,23,53] and based on data obtained using the Delphi method (described above).

The primary data were analysed quantitatively, enabling the description of general trends, the identification of differences between selected groups of respondents, and the identification of latent factor structures. The main tools of statistical analysis were measures of central tendency, i.e., the arithmetic mean and median, which made it possible to determine the mean and median scores assigned to individual variables influencing purchasing decisions on the dairy market [55]. Using a ranking procedure involving the assignment of ordinal numbers (ranks) to arithmetic mean scores, a hierarchy of variables could be created according to their strength of influence on purchasing decisions [56].

In order to conduct an in-depth assessment of the distribution of variables, measures of asymmetry (skewness) and kurtosis were also used, which allowed for the assessment of the shape of the response distribution and verification of the validity of using non-parametric tests. The normality of the distribution of the studied variables was also checked using the Kolmogorov–Smirnov test with Lilliefors correction. The test results indicate that for all analysed variables, statistically significant test values (p < 0.001) were obtained, which means that the distributions of the studied variables deviate significantly from the normal distribution. The statistical description was complemented by measures of dispersion, namely the standard deviation and the coefficient of variation, which allowed the degree of variation in the responses provided by the study group to be assessed [57]. To identify differences in responses between men and women, comparative analysis (using measures of central tendency and dispersion in analysed groups), the Mann–Whitney U test, and exploratory factor analysis (EFA) using the principle components (PCA) method were applied. The non-parametric Mann–Whitney U test was used to test for differences between two independent groups. This test is used when the dependent variable is measured at least on an ordinal scale, but the analysed data do not meet the assumptions required for parametric tests. The Mann–Whitney U test involves ranking the dependent variable scores (from smallest to largest) in the groups under study. The statistic for testing the null hypothesis of no difference between groups is to compare the mean of the ranks for each of the two groups and determine whether this difference is statistically significant at the assumed significance level of p ≤ 0.05 [58]. The test results were presented in the form of the U statistic value corresponding to its significance level (p) and the means and sums of ranks for the analysed groups. For a more in-depth interpretation of the results obtained, the effect size (r) was also calculated, which allowed for an assessment of the strength of the differences between the groups of respondents [59].

Exploratory factor analysis (EFA) was performed using the principle components method to reduce the number of observable variables by transforming a system of mutually correlated variables into a new system of mutually uncorrelated, yet comparable, variables. The extracted factors are assumed to reach a ‘deeper’ level of the reality being studied and represent the underlying causes of the observable variables. The advantage of exploratory factor analysis is, therefore, the ability to discover the optimal number of latent factors that explain the relationships between observable variables [60]. Before proceeding with the actual analysis, the reliability of the measurement scale was checked using Cronbach’s alpha coefficient, which enabled the assessment of the internal consistency of the questionnaire items. Preliminary tests were also conducted to assess the suitability of the data: Bartlett’s sphericity test and the Kaiser–Meyer–Olkin (KMO) measure of sample adequacy. A KMO value above 0.7 and a Bartlett test significance of p < 0.001 confirmed the possibility of conducting EFA (Hair et al., 2019). Exploratory factor analysis was employed to reduce the set of variables influencing the research category ‘factors influencing dairy purchasing decisions’, and to detect internal correlations in the relationships between these variables.

In order to identify the number of factors, i.e., the number of strongly correlated variables (so-called principal components), the following criteria were used: Kaiser’s criterion, Cattell’s criterion and the criterion of factor interpretability from the point of view of the adopted research design. For the total number of respondents, the number of factors was determined using the Kaiser method, and for groups of respondents separated by gender, the Cattell method was used. According to the Kaiser criterion, only factors with eigenvalues greater than 1 are left for further analysis, while according to the Cattell scatterplot criterion, those lying on the ‘slope’ on the line graph, i.e., to the left of the factor scatterplot, together with the principal component from which the scatterplot starts, are left for further analysis [61]. Each factor – identified in the exploratory factor analysis process – explains a certain level of the phenomenon’s overall variability, defined by a percentage of variance, which can be interpreted as a measure of its explanatory power. The factors were rotated using the Varimax method. Within individual factors, variables with the highest factor loadings were identified in relation to a given factor, i.e., variables with values of at least 0.5, which is the limit commonly accepted in the literature [62]. The results of the exploratory factor analysis are presented in a table containing eigenvalues and the percentage of variance explained by each factor, as well as in a table presenting the structure and content of the identified factors (motivational segments), together with the variables that constitute them and their factor loadings – separately for the total sample, women and men. This approach allowed for conclusions to be drawn about the empirical and interpretative significance of the factors obtained. Statistical analysis of the collected primary data was performed using PS IMAGO PRO 10.0 (IBM SPSS Statistics 29).

## 3. Research findings

### 3.1 Factors influencing purchasing decisions on the dairy market

Respondents were presented with a list of 26 motives for purchasing dairy products and asked to rate their importance on a 5-point Likert scale (Table 1). Of the 26 motives studied in terms of their influence on purchase decisions, three were related to the health-promoting qualities of the product (product composition, health values and nutritional values). Five were related to the quality of the product, including product safety (shelf life, lack of preservatives and a quality certificate), organic/bio products and sensory qualities (taste and smell). Another five motives were related to the origin and trustworthiness of the product (product brand, producer, country of origin and whether it is a local product). Four motives were related to economic and practical qualities (price, pack size, income level and shop availability). Five variables were related to marketing determinants and their impact at the point of sale (commercial promotions, display, loyalty programmes, tasting, and packaging appearance). Four determinants were related to social and psychological issues (habit, family member preference, fashion and curiosity about new products).

**Table 1:** Motivations for purchasing decisions in the dairy market among total respondents.

Variables	% of respondents					Me	$\overset{―}{𝐗}$	SD	V	Rank
	1	2	3	4	5					
Composition of the product	0.5	5.5	11.9	28.9	53.2	5	4.3	0.915	21.3	1
Health values	1	4.5	14.4	33.8	46.3	4	4.2	0.917	21.8	2
No preservatives	1.5	5.5	13.9	30.8	48.3	4	4.2	0.972	23.2	3
Nutritional values	1	4.5	16.4	33.8	44.3	4	4.2	0.924	22.2	4
Sensory properties (taste. smell)	2	4.5	14.9	37.3	41.3	4	4.1	0.955	23.2	5
Shelf life	2.5	5	21.4	32.3	38.8	4	4	1.015	25.4	6
In-store availability	1.5	4.5	21.4	42.3	30.3	4	4	0.913	23.1	7
Habits	3.5	4.5	18.9	48.8	24.4	4	3.9	0.954	24.7	8
Quality certificate	4.5	6	25.4	33.8	30.3	4	3.8	1.079	28.4	9
Country of origin of the product	4	11.4	19.9	33.8	30.8	4	3.8	1.128	30	10
Preference of family members	3.5	8	19.9	47.3	21.4	4	3.8	0.994	26.5	11
Local product	3.5	10.9	17.9	43.8	23.9	4	3.7	1.051	28.1	12
Traditional recipes	3.5	9	25.4	40.3	21.9	4	3.7	1.024	27.8	13
Price	3.5	8	29.9	36.3	22.4	4	3.7	1.022	27.9	14
Pack size	3.5	11.4	30.3	38.3	16.4	4	3.5	1.010	28.6	15
Product brand	5.5	9.5	32.8	37.8	14.4	4	3.5	1.029	29.7	16
Manufacturer	4	11.9	35.8	34.8	13.4	3	3.4	0.997	29.2	17
Curiosity about a new product	5.5	14.4	29.9	33.3	16.9	4	3.4	1.097	32.1	18
Organic/bio product	6.5	17.4	24.4	33.3	18.4	4	3.4	1.162	34.2	19
Income level	8.5	11.9	33.3	28.4	17.9	3	3.4	1.157	34.5	20
On-site sales promotions	10	19.9	29.4	27.4	13.4	3	3.1	1.181	37.6	21
Display at point of sale	12.4	15.4	38.3	24.9	9	3	3	1.124	37.2	22
Packaging appearance	10.9	19.9	32.8	29.4	7	3	3	1.102	36.6	23
Loyalty programmes	14.9	20.4	37.3	21.9	5.5	3	2.8	1.102	39	24
On-site tastings	24.2	24.9	22.9	18.9	9	3	2.6	1.282	48.7	25
Product fashion	26.4	21.9	31.8	12.9	7	3	2.5	1.209	47.9	26

Symbol designations: Me – median, $\overset{―}{\text{X}}$ – arithmetic mean, SD – standard deviation, V – coefficient of variation.

Rating scale: 1 – not important at all, 2 – rather not important, 3 – hard to say, 4 – rather important, 5 – very important.

The ranking of themes was based on arithmetic means.

Source: Results of own research.

In the context of the Theory of Planned Behaviour (TPB), above mentioned factors constitute an element of:

- Attitudes toward the behaviour, which refer to the assessment of a purchase as beneficial or disadvantageous and result from beliefs about the properties of the product and the benefits of consuming it. These are the following motivators: product composition, health benefits, no preservatives, nutritional value, sensory properties (taste, smell), quality certificate, country of origin, local product, traditional recipes, product brand, manufacturer, curiosity about a new product, organic/bio product.
- Subjective norms, which result from social pressure or the expectations of those around us, e.g., family, friends, professional environment. These motivators include: habits, family members’ preferences, product trends.
- Perceived behavioural control, which refers to the ease or difficulty of making a purchase, taking into account external factors such as availability, time, financial resources and market supply. These are the following motivators: expiry date, availability in the shop, price, package size, income level, sales promotions at the point of sale, display at the point of sale, loyalty programmes, tastings at the point of sale.

The arithmetic mean for the aforementioned motives ranges from 2.5 to 4.3. Sorting the mean values produced a hierarchy of variables, taking into account their strength of influence on purchasing decisions. This procedure made it possible to identify four groups of motives. The main motives the surveyed doctors declared that influenced their purchasing decisions for dairy products, were product composition, health values, the absence of preservatives, nutritional values, sensory values, shelf life and availability in shops. The mean score values for these seven variables were at least 4.0. Each motive in this group was considered important or very important in the decision-making process by more than 72% of the health service respondents. As many as four-fifths of respondents – medical practitioners – declared that they are guided by product composition in their decision-making process, and this variable received the highest average value of 4.3. It was considered very important by more than 53% of respondents. These results suggest that, when buying dairy products, doctors are most often guided by health and quality values as well as pragmatic ones, such as shelf life and availability. Therefore, the most important purchasing motives in the dairy market among the doctors surveyed are quality and health-related. This means that doctor-consumers prefer dairy products that are perceived as natural and safe for health, that taste good and that are easily available. Our research findings demonstrate that, in the context of the Theory of Planned Behaviour, attitudes towards product quality and health, together with perceived behavioural control related to availability, play a decisive role in purchasing intentions. Similar results were obtained in previous studies based on the TPB model [63].

The second group of motives influencing doctors’ purchasing decisions in the dairy market has an average score ranging from 3.5 to 3.9. This group includes nine variables indicating dietary habits and preferences of family members, food safety aspects such as quality certification, product country of origin, local products and traditional recipes, product brand, as well as economic and practical aspects such as price and pack size. These motivations are important to between 52% and 73% of respondents. Motivators with a moderate but still significant influence on doctors’ purchasing decisions are those indicating a preference for products of known origin and safety, as well as economic factors.

The third group comprises motivators related to product qualities such as ‘new to the market’ and ‘organic/bio products’, as well as marketing activities (e.g., trade promotions and displays at the point of sale, packaging appearance, manufacturer). The arithmetic mean of these variables was between 3.0 and 3.4. Individual motivators were important in purchasing decisions for 40% to 52% of doctors surveyed. Therefore, market novelties, organic products, marketing activities, and the material situation of purchasers from a medical profession are of limited importance in dairy market purchasing decisions.

According to the surveyed doctors, the weakest motivational functions in relation to the purchase decision of dairy products are loyalty programmes ($\overset{―}{\text{X}}$ = 2.8) and tastings at the point of sale ($\overset{―}{\text{X}}$ = 2.6), which are important for around a quarter of respondents, and product fashion ($\overset{―}{\text{X}}$ = 2.5), which motivates less than a fifth of respondents. This may indicate that Polish buyer-doctors in the dairy market are rather conservative and do not pay much attention to marketing activities undertaken at points of sale.

It is worth noting that the standard deviation value for the first 18 variables did not exceed one-third of the mean value, as can be seen from the fact that more than 70% of observations cluster around the mean. The consumer-doctors’ opinions regarding the key purchase motives are consistent and not random. Fewer than 70% of observations cluster around the mean for the remaining eight variables. This indicates a dispersion of respondents’ evaluations and justifies using the median as a more appropriate measure of central tendency. As the middle value in an ordered dataset, the median divides it into two equal parts and better reflects the nature of the distribution of the more dispersed variables. In the case of the ‘environmental product’ variable, the median value was Me = 4, whereas for the other variables it was Me = 3.

The analyses revealed that H1 was empirically confirmed in the studied group of doctors: purchasing decisions in the dairy market are multidimensional, shaped by diverse motives including health and quality rationales related to nutritional safety, economic and pragmatic determinants related to origin and trust in the product, and, to a lesser extent, marketing activities.

To summarise this point, it should be concluded that the first hypothesis (H1), according to which the purchasing decisions of the surveyed final consumers represented by Polish doctors on the dairy products market are shaped by various determinants, should be accepted as true.

### 3.2 Motives for purchasing dairy products and the gender of respondents

The next analysis stage involved comparing the ratings of variables influencing purchasing decisions in the dairy market among women and men. This comparison aimed to identify any differences in the hierarchy of variables influencing purchasing decisions between the two gender groups. Looking at the hierarchy of variables ratings, some differences can be seen in the responses of women and men (see Table 2). The variables with the highest ratings for female doctors are product composition, health values, nutritional values, lack of preservatives and sensory properties (taste and smell), with an arithmetic mean between 4.16 and 4.38. The highest rated variables for male doctors are product composition, shelf life, lack of preservatives and sensory properties, with an arithmetic mean between 4.02 and 4.15. By comparing these hierarchies, we can conclude that, while both women and men value product composition, lack of preservatives and sensory properties the most, women place greater importance on pro-health and nutritional aspects, while men prioritise the practical dimension of products, such as shelf life. Comparing the evaluations of individual purchase determinants by women and men (Table 2) revealed that women’s evaluations of 26 variables had a higher arithmetic mean than men’s: ranging from 2.6 to 4.38 for women and from 2.41 to 4.15 for men. Analysis of the arithmetic means indicates that, for 22 of the studied variabless, the values were higher among the female doctors, while for only four variables was the mean value higher among the male doctors. The most significant differences in mean values between male and female respondents were recorded for motives such as organic/bio products (0.44), producers (0.37), quality certificates (0.34), tasting at the point of sale (0.32) and health values (0.30). In these cases, the female group’s average was higher than the male group’s. These results suggest that the analysed purchasing motives were more important to female respondents than to male respondents. Motives for which the mean values were higher among men included shelf life (0.18), package size (0.11), country of origin (0.03), and trade promotions at the point of sale (0.03).

**Table 2:** Motivations for purchasing decisions in the dairy market and gender (%).

	Female		Male		Difference in means (F-M)	Rank for total
	$\overset{―}{𝐗}$	Rank	$\overset{―}{𝐗}$	Rank		
Product composition	4.38	1	4.15	1	0.23	1
Health values	4.32	2	4.02	5	0.3	2
No preservatives	4.25	4	4.1	3	0.15	3
Nutritional values	4.27	3	4	6	0.27	4
Sensory properties (taste. smell)	4.16	5	4.05	4	0.11	5
Shelf life	3.93	7	4.11	2	−0.18	6
Availability in shop	3.98	6	3.93	7	0.05	7
Habit	3.91	9	3.79	8	0.12	8
Quality certificate	3.93	8	3.59	12	0.34	9
Country of origin of the product	3.75	12	3.78	9	−0.03	10
Preference of family members	3.83	10	3.63	10	0.2	11
Local product	3.82	11	3.62	11	0.2	12
Traditional recipes	3.74	13	3.59	13	0.15	13
Price	3.71	14	3.59	14	0.12	14
Pack size	3.48	19	3.59	15	−0.11	15
Product brand	3.54	17	3.35	16	0.19	16
Manufacturer	3.57	16	3.2	19	0.37	17
Curiosity about a new product	3.51	18	3.28	17	0.23	18
Organic/bio products	3.58	15	3.14	21	0.44	19
Income level	3.44	20	3.22	18	0.22	20
On-site sales promotions	3.13	21	3.16	20	−0.03	21
Display at point of sale	3.07	22	2.96	23	0.11	22
Packaging appearance	3.02	23	3.01	22	0.01	23
Loyalty programmes	2.85	24	2.79	24	0.06	24
Tasting at point of sale	2.76	25	2.44	25	0.32	25
Product fashion	2.6	26	2.41	26	0.19	26

Symbol designations: $\overset{―}{\text{X}}$ – arithmetic mean, F- female, M – male.

Rating designations: 1 – not important at all, 2 – rather not important, 3 – hard to say, 4 – rather important, 5 – very important.

Source: Own study.

Considering the differences in the average assessment of the motivators of purchasing decisions between men and women, it should be noted that despite the similarities in the hierarchy of values of those surveyed, differences can be observed in the assessment of the importance of individual variables. While both groups of doctors emphasised the importance of product composition, the absence of preservatives and sensory properties in their choices, women placed greater importance on health-enhancing and nutritional aspects, while men prioritised practical aspects.

The next step was to try to answer the question of whether there was a statistically significant difference between male and female doctors in their assessment of the motivational strength of these variables. For this purpose, a Mann-Whitney U test was performed (see Table 3).

**Table 3:** Significance analysis of differences between respondents’ answers by gender.

Variables	Gender	Mean rank	Sum of ranks	Mann-Whitney U	Z	R	Asymptotic significance (two-sided)
Health values	F	108.76	13051	3929	−2.48	0.18	**0.013**
	M	89.51	7250				
Nutritional values	F	107.2	12863.5	4116.5	−1.97	0.14	**0.049**
	M	91.82	7437.5				
Shelf life	F	97.97	11756.5	4496.5	−0.95	0.07	0.343
	M	105.49	8544.5				
Product composition	F	107.24	12868.5	4111.5	−2.04	0.14	**0.041**
	M	91.76	7432.5				
Sensory properties (taste. smell)	F	103.49	12418.5	4561.5	−0.79	0.06	0.43
	M	97.31	7882.5				
No preservatives	F	104.91	12589	4391	−1.25	0.09	0.21
	M	95.21	7712				
Organic/bio products	F	109.72	13166	3814	−2.67	0.19	**0.008**
	M	88.09	7135				
Quality certificate	F	109.72	13166	4034	−2.13	0.15	**0.033**
	M	88.09	7135				
Traditional recipes	F	105.04	12604.5	4375.5	−1.26	0.09	0.209
	M	95.02	7696.5				
Manufacturer	F	109.04	13084.5	3895.5	−2.50	0.18	**0.012**
	M	89.09	7216.5				
Country of origin of the product	F	100.69	12083	4823	−0.10	0.01	0.924
	M	101.46	8218				
Local product	F	104.72	12566.5	4413.5	−1.17	0.08	0.243
	M	95.49	7734.5				
Availability in shop	F	102.75	12330	4650	−0.55	0.04	0.581
	M	98.41	7971				
On-site tastings	F	106.43	12772	4208	−1.65	0.12	0.098
	M	92.95	7529				
Loyalty programmes	F	102.1	12251.5	4728.5	−0.34	0.02	0.735
	M	99.38	8049.5				
Display at point of sale	F	102.99	12358.5	4621.5	−0.61	0.04	0.539
	M	98.06	7942.5				
On-site sales promotions	F	100.43	12051	4791	−0.18	0.01	0.861
	M	101.85	8250				
Price	F	103.6	12432	4548	−0.81	0.06	0.42
	M	97.15	7869				
Product brand	F	105.4	12648	4332	−1.37	0.10	0.17
	M	94.48	7653				
Pack appearance	F	101.41	12169.5	4810.5	−0.13	0.01	0.899
	M	100.39	8131.5				
Pack size	F	98.65	11838	4578	−0.73	0.05	0.465
	M	104.48	8463				
Income level	F	104.71	12565.5	4414.5	−1.14	0.08	0.254
	M	95.5	7735.5				
Product fashion	F	104.54	12544.5	4435.5	−1.,08	0.08	0.278
	M	95.76	7756.5				
Habits	F	103.57	12428.5	4551.5	−0.82	0.06	0.412
	M	97.19	7872.5				
Curiosity about a new product	F	104.7	12564	4416	−1.14	0.08	0.255
	M	95.52	7737				
Preference of family members	F	105.68	12681.5	4298.5	−1.48	0.10	0.138
	M	94.07	7619.5				

Symbol designations:: F-female, M-male.

Source: Own elaboration.

The non-parametric Mann-Whitney U test, conducted at a significance level of p ≤ 0.05, showed statistically significant differences between men and women in assessing the motivational strength of the importance of 6 of the 26 variables considered in the purchasing decision-making process in the dairy market. In each of these cases, the effect size (r) was small, ranging from 0.1 to 0.3. Therefore, research hypothesis H2, which states a gender difference in the assessment of the importance of individual motivators in purchasing decisions among the surveyed doctors, is true for 6 of the 26 variables. The motivators in question are: health values, nutritional values, product composition, organic products, quality certificates and producers. In all cases, women’s average rank is higher than men’s, indicating that these motivators are more important for women than for men.

To summarise the considerations in this section, it should be noted that despite the limited diversity of responses from the women and men surveyed in the analysed context, hypothesis two (H2) can only be considered true to a certain extent.

### 3.3 Latent factors influencing purchasing decisions

To determine the optimal number of latent factors that explain the correlation structures between the observable variables, ‘factors influencing dairy purchase decisions’, an exploratory factor analysis (EFA) was conducted for all respondents and for the categories identified based on the demographic variable of gender. Principal component analysis was then used to extract the main factors. Prior to the analysis, the data were standardised.

First, Cronbach’s alpha coefficient was calculated to assess the reliability of the measurement tool. The catalogue of 26 analysed variables relating to purchase motives reached alpha coefficient values of 0.925 for the total sample, 0.934 for women and 0.909 for men, indicating very high reliability and internal consistency of the scale. In the next step, communality values were calculated, which indicate the extent to which the variability of individual variables is explained by the identified factors. The results obtained range from 0.55 to 0.74, which means that most of the variables were well represented by the factor model. The highest communality values were obtained for the variables: product brand (0.737), no preservatives (0.709) and price (0.703), which indicates their strong correlation with the identified factors. Slightly lower values (approx. 0.55) were recorded for variables such as family member preferences and loyalty programmes, but these also exceed the minimum acceptable threshold of 0.40, confirming the adequacy of the items included in the model.

Next, an EFA using the principle components analysis method (PCA) was carried out for the total sample and the sex-disaggregated categories. This resulted in extracting five latent factors for each analysed category of respondents (total, female and male). For the entire sample, the number of factors was determined using the Kaiser criterion, whereby only factors with an eigenvalue greater than 1 were included. Conversely, in the analyses conducted separately for women and men, the Cattell criterion was applied based on scree plot analysis. In this approach, the factors lying above the ‘scree plot’, i.e., the point of inflection of the curve indicating the borderline of significant components in the line plot, were extracted.

In each case, the extracted latent factors explain more than 62% of the total variability (the highest cumulative eigenvalue is found in the female category at 66%), which indicates a good model structure (see Table 4). The Kaiser-Meyer-Olkin (KMO) index, which is used to measure the adequacy of variable selection, exceeded the recommended threshold of 0.7 for all analysed categories, indicating the validity of using exploratory factor analysis with principal component method. Bartlett’s test of sphericity was statistically significant in all extracted categories (p < 0.001), confirming the existence of relationships between variables and the validity of the analysis.

**Table 4:** Hierarchy of factors according to their eigenvalues determined using the Kaiser criterion for all respondents and the Cattell criterion for women and men.

Main components	eigenvalue			cumulative eigenvalue			% of total eigenvalues (variance)			cumulative % of eigenvalues		
	T	F	M	T	F	M	T	F	M	T	F	M
1	9.18	9.91	8.13	9.18	9.91	8.13	19.92	20.81	20.29	19.92	20.81	20.29
2	3.14	3.27	3.49	12.31	13.17	11.62	16.33	17.65	11.54	36.26	38.46	31.83
3	1.60	1.83	1.73	13.91	15.00	13.34	9.66	10.85	11.53	45.92	49.31	43.37
4	1.28	1.22	1.57	15.20	16.22	14.92	9.20	8.91	11.45	55.12	58.22	54.81
5	1.04	0.94	1.36	16.23	17.17	16.27	7.32	7.81	7.78	62.44	66.02	62.59

Symbol designations: T – total, F – female, M – male.

“Total” - Kaiser-Meyer-Olkin (KMO) measure of sampling adequacy is 0.904, which is greater than 0.7. Bartlett’s sphericity test is significant (variables are statistically significantly related); chi^2^ = 2624.309;

and p < 0.001;

“Female” – KMO = 0.871; Bartlett’s test of sphericity is significant; chi^2^ = 1856.993; and p < 0.001;

“Male” - KMO = 0.814; Bartlett’s test of sphericity is significant; chi^2^=1101.656; p<0.001.

Source: Own elaboration.

Factor 1 achieved very high consistency (α = 0.897 for the total, 0.899 for women, 0.897 for men), which indicates that the variables forming this factor are strongly correlated and measure the same latent trait. Factor 2 showed good consistency overall and in the group of women (α = 0.856), while in men the consistency was moderate (α = 0.722). Factor 3 is characterised by acceptable consistency for the total sample and for women (α = 0.758 total, 0.717 women), and good consistency for men (α = 0.819 men). Factor 4 achieved α values within acceptable limits (α = 0.774 overall, 0.704 women, 0.773 men), which allows it to be considered a psychometrically reliable factor. Factor 5 shows varied consistency: acceptable for the total (α = 0.727), low for women (α = 0.619) and very low for men (α = 0.493). This means that the variables in this factor poorly measure the same trait, so its interpretation should be treated as preliminary and requires further verification in confirmatory studies.

When the internal structure of each factor was analysed, clear differences were observed between female and male respondents, as well as between each category and all respondents. Each factor represents a group of characteristics that jointly explain part of the variability in responses. High factor loadings indicate which variables are dominant in a given factor.

Five factors were identified for each category of respondents analysed, based on the motives determining purchase decisions in the dairy market and interpreted in terms of consumer profiles (Table 5).

**Table 5:** Segments of respondents distinguished by their purchase decision motives in the dairy market (for total respondents and women and men).

Total	MOTIVATION PRO-HEALTH Lack of preservatives (0.819) Health values (0.755) Organic/bio product (0.754) Quality certificate (0.724) Product composition (0,720) Traditional recipes (0,707) Local products (0.693) Nutritional values (0.642)	ATTRACTIVENESS MARKETING Product fashion (0.791) Tasting at point of sale (0.720) Display at point of sale (0.709) Packaging appearance (0.702) Loyalty programmes (0.646) Sales promotions at point of sale (0.625)	HABITS AND CONSUMER RECEPTIVENESS Habits (0.792) Family preferences (0.595) Sensory attributes (0.542) Curiosity about the new product (0.514)	ATTRACTIVENESS PRAGMATIC-ECONOMIC PURCHASE Price (0.707) Packaging size (0.681) Income level (0.589) In-store availability (0.571)	GUARANTEE OF PRODUCT QUALITY AND ORIGIN Shelf life (0.605) Product brand (0.543) Manufacturer (0.512) Country of origin of the product (0.505)
Women	HEALTH MOTIVATION No preservatives (0.832) Composition of the product (0.764) Organic/Bio product (0,739) Quality certificate (0.732) Local produce (0,720) Health values (0,708) Nutritional values (0,691) Traditional recipes (0.601) Country of origin of the product (0.537)	MARKETING AND PROMOTIONAL APPEAL Product fashion (0.819) Tasting at point of sale (0.798) Display at point of sale (0.758) Trade promotions at point of sale (0.738) Loyalty programmes (0.707) Appearance of packaging (0.679)	PRAGMATIC AND ECONOMIC ATTRACTIVENESS OF THE PURCHASE Price (0.753) Level of income (0.746) Package size (0.644)	REPUTATION AND SAFETY Product brand (0.708) Manufacturer (0.652) Shelf life (0.601)	HABITS Habits (0.822) Family members’ preferences (0.585)
Men	HEALTH MOTIVATION No preservatives (0.816) Health values (0.808) Traditional recipes (0.768) Organic/bio product (0.722) Quality certificate (0,713) Composition of the product (0.711 Local product (0,658) Nutritional values (0.610)	CONSUMER HABITS AND RECEPTIVENESS Habituation (0.760) Sensory properties (taste, smell)(0.699) Curiosity about the new product (0.631)	MARKETING AND IMAGE APPEAL Appearance of packaging (0.787) Product fashion (0.658) Product brand (0.631) Manufacturer (0.570) Display at point of sale (0.535)	ECONOMIC AND PROMOTIONAL APPEAL Trade promotions at point of sale ((0.664) Price (0.643) Loyalty programmes (0.589) In-store availability (0.558) Revenue level (0.534)	PRAGMATIC QUALITIES OF THE PRODUCT Shelf life (0.772) Pack size (0.509)

Source: Own study.

The first principal component for all categories of respondents indicates health-promoting motivations, suggesting that consumers with a medical background primarily base their purchasing decisions on the health benefits, quality, and natural character of dairy products. Although the hierarchy of individual variables varies slightly across the analysed female and male doctors, the structure of the first factor remains stable, confirming its universal nature.

Despite differences in the load hierarchies of individual variables, the second principal component is universal for total and female respondents. It can be described as the marketing attractiveness of the product and indicates decision-making influenced by consumer trends created by marketing specialists, marketing activities undertaken at the point of sale, and packaging aesthetics. The second factor in the male group differs significantly from that in the other analysed groups. It suggests that men’s purchasing decisions are influenced by routine and openness to novel sensory experiences.

The third principal component is characterised by significant variation in purchasing motivations across the different groups of respondents. For all respondents, purchasing decisions are primarily shaped by established consumer habits and the preferences of family members. Among the total respondents, purchase decisions are also motivated by sensory attributes and openness to novelty. For women, the factor reflects a strong orientation towards the economic aspects of purchasing dairy products decision. In this group, the highest factor loadings were given to variables relating to price, pack size and income level, indicating rational purchasing linked to an assessment of the profitability of the purchase. For men, marketing image attractiveness is emphasised, including the appearance of the packaging, fashion trends, product branding, manufacturer and display at the point of sale.

The fourth principal component reveals a distinct structure that varies depending on the category of respondents analysed. The results of the exploratory factor analysis show that economic aspects (price and income level) play a dominant role for all the respondents, as do pragmatic aspects (package size and product availability). This suggests that purchasing decisions are largely influenced by economic and pragmatic motivations related to assessing the profitability of the purchase and the product’s availability. For female respondents, however, this factor takes on a different character. The key variables are product brand, manufacturer and expiry date, indicating a high concern about food security. For men, promotional aspects are dominant, as indicated by high loadings for factors such as in-store commercial promotions, price, loyalty programmes and in-store availability. This, therefore, indicates their susceptibility to marketing activities.

An apparent variation in the structure of the principal component according to the analysed category is also revealed for the fifth factor. For all respondents, the fifth principal component indicates a strong focus on food security, as evidenced by the high factor loadings of variables such as shelf life, product brand, producer and country of origin. For women, however, this factor is different, focusing on consumption routines determined by the consumption habits of family members. This may imply decision-making based on established patterns and the household’s needs. For men, the fifth latent factor structure indicates dairy products’ pragmatic utility, with variables such as shelf life and package size being loaded.

EFA reveals that the factors shaping purchase decisions differ significantly between men and women with a medical background, confirming research hypothesis H3 that there are gender-based differences in the latent factors determining purchase decisions. While the first main component, health-promoting motivation, is common to both genders, there are noticeable differences in the composition and hierarchy of the remaining latent factors. These differences suggest that the determinants of purchasing decisions are gender-specific.

The variation in factor structure extracted through exploratory factor analysis using the principal components method indicates significant differences in the determinants of respondents’ purchasing decisions. These differences have important implications for marketing and communication strategies in the dairy market. Market segmentation should consider not only gender, but also dominant decision-making motivations (see Table 5).

Referring to hypothesis three (H3), according to which there is a difference in the structure of hidden factors influencing purchasing decisions in the dairy products market depending on the gender of the doctors surveyed, it should be considered true.

## 4. Discussion

It is difficult to directly relate the obtained research results to other authors due to the existing research gap. However, it is possible to compare them to selected studies relating to food products or the studied product group based on other consumer groups’ opinions.

A survey conducted among doctors in Poland showed that the composition of the product and health-promoting factors play a key role in the decision to purchase dairy products, as indicated by the high values of the measures of central tendency (arithmetic mean and median) and the low coefficient of variation obtained in the responses of the surveyed respondents.

In practice, this means a preference for dairy products that are perceived as good for health, high nutritional value, and free of artificial additives. These results are consistent with findings in the literature. For example, surveys of general consumers have shown that up to 69% of respondents primarily consider health when purchasing dairy products [64]. Notably, the surveyed doctors deemed health and quality more important than economic factors. This is consistent with the general trend observed among more discerning consumers. Chudzian [65] found that, when purchasing dairy products, consumers most frequently select products based on quality, composition and health values, while price is less important. Quality-oriented consumers are willing to pay more for products that meet their health expectations, and this attitude was also evident among the surveyed doctors, for whom nutritional safety issues dominated over purely economic considerations.

Although health factors dominated the responses of the surveyed doctors, the sensory properties of dairy products cannot be underestimated. Even among medical professionals with expertise in nutrition, the taste and smell of dairy products remain important motivators for purchase. Both subgroups of respondents (women and men) emphasised the importance of taste, smell, and appropriate product composition. This is consistent with the findings of numerous market studies, which have consistently identified taste as a key factor in food choices, including dairy products. For instance, Angowski et al. [66] discovered that taste (and its associated smell) was the primary factor influencing dairy purchases among young consumers, surpassing other attributes. Furthermore, attributes indicative of quality, such as freshness or shelf life, were ranked just behind sensory qualities by young purchasers, while health aspects were ranked even further ahead. However, our results suggest that, although the doctors surveyed value taste, they do not prioritise sensory pleasure over health benefits. In other words, while taste is an important condition for product acceptance, they prioritise fulfilling health criteria. This hierarchy may distinguish doctors from the average consumer. The typical consumer often chooses dairy products primarily for taste and enjoyment. In contrast, doctors are more willing to compromise on taste if the product offers higher nutritional or health value. This trend is supported by the observation that those with greater nutritional knowledge are more likely to perceive food quality holistically – considering not only the sensory experience, but also the composition and potential health benefits.

The literature shows that, in recent years, many consumers have become more environmentally conscious and sensitive to how and where food is produced [67]. Some consumers prioritise including sustainably produced products in their diet and are willing to pay more for such products [68]. However, the research presented in this study has shown that organic products play a limited role in purchasing decisions in the dairy market. Therefore, taking active measures to increase the consumption of this group of products seems justified, both in the context of sustainable consumption goals and for health reasons.

A study by Grebowiec [11] shows that sensory and functional impressions, as well as the best-before date, are the most important factors influencing the purchase of dairy products. These results are therefore partly in line with those obtained in the present study, in which sensory impressions were found to be an important factor in the decision to purchase, albeit not the most important one. In his earlier study, Grebowiec [10] showed that many of the products analysed are purchased routinely. However, in that study, sensory impressions, expiry date and brand loyalty were the main determinants of purchase of these products. The research presented here confirms that brand and expiry date are moderately influential factors in purchasing decisions. The quality factor is notable due to its proven importance as a purchase determinant in the dairy market. Sajdakowska et al. [20] found that consumers generally perceive food quality based on attributes such as freshness, naturalness and production method, as well as appearance, taste and smell. However, when it comes to food of animal origin, convenience related to availability, nutritional value and health benefits are the most important factors. In a survey of young consumers, Gaworski et al. [2] highlighted the importance of taste and quality of dairy products, and the minor role of packaging in their purchase.

It was found that price and marketing activities were less influential in doctors’ purchasing decisions than health and quality aspects. Respondents showed high levels of resistance to marketing influences – motives such as product tastings at the point of sale or product popularity trends were rated the lowest. Doctors therefore adopt a rational and conservative approach, making decisions based on the objective qualities of the product rather than the advertising message. It is also worth noting that, in the general population, consumers declare limited susceptibility to typical marketing stimuli. The research shows that 57.5% of respondents claim that advertising does not influence their dairy choices and less than 4% admit to being influenced by advertising. Furthermore, over 60% of respondents do not trust advertising messages for food products [64]. While the actual influence of marketing is often unconscious, these stated attitudes suggest that doctors, like many other consumers, do not want to see themselves as susceptible to marketing.

In terms of economic factors, the doctors surveyed considered the product’s price to be of moderate importance. Price was a consideration, but not a dominant factor, which differs from some mass-market segments. By contrast, nationwide surveys indicate that the average consumer considers the price and brand of a product to be important criteria, although these tend to be ranked just behind quality and taste in the hierarchy [64,65]. For instance, one survey found that the average importance of price when purchasing dairy products was 3.32 out of 5, while quality and brand were rated at 3.48 and 3.40 respectively [64]. This demonstrates the moderating role of price: important, but not always decisive. The doctors we surveyed emphasised price more than product brand or the manufacturer’s reputation. At the same time, they rated quality certificates highly, distinguishing them from the average consumer. As a group with a relatively stable financial situation and high awareness, doctors may be less sensitive to minor price differences if they gain a higher-quality or healthier product in return.

The research presented in this study showed that gender influenced the motives behind purchasing decisions in the dairy market among the doctors surveyed. Kurajdová et al. [69] demonstrated that, in the Slovak market, gender was a predictor, albeit a relatively weak one, of milk purchasing motives. A study conducted in the Czech Republic by Hrubá and Sudzina [70] found that nutritional value, manufacturer, product origin and online information were significantly gender-dependent. Women pay more attention to the nutritional value and manufacturer of the products they buy, and look for desirable information on labels more often than men do. Conversely, men pay more attention to the product’s origin and information about the product on the internet. Szwacka-Mokrzycka et al. [71] demonstrated in their study that gender also influences the information on food packaging. All packaging attributes except size (e.g., ease of handling/transportation, ease of opening, aesthetics, and recycling potential) were considered more important by women than by men.

The study results form part of the broader context of consumer behaviour in the food market. The literature highlights an increasing interest in functional and healthy foods, as evidenced by the popularity of enriched dairy products such as probiotics and cholesterol-lowering foods [65]. As consumers with above-average health consciousness, doctors are likely to be among the trendsetters in this area. They can also influence their patients’ and families’ attitudes through recommendations and personal examples. Their rational, knowledge-based approach to purchasing dairy products can therefore influence the attitudes of a wider range of consumers through recommendations and personal example. This suggests to dairy producers and retailers the need to segment the market and develop offers and messages for health-conscious groups (such as doctors) and groups driven mainly by taste or price. Indeed, as segmentation analyses of student consumers show, the market comprises very different groups – from those with an environmental and quality orientation, to those driven almost exclusively by taste, freshness, and price.

## 5. Conclusions

In summary, doctors’ purchasing decisions in the dairy market are multidimensional and based on various motivations. Hypothesis H1 on the multifactorial nature of choices has been fully confirmed – even informed consumers such as doctors take health and quality into account, as well as economic, psychosocial (e.g., habits) and marketing-related factors, although the latter play a secondary role. This profile of the medical consumer/buyer means that food policy institutions and dairy producers should primarily emphasise the health benefits, naturalness and high quality of their products to meet this group’s expectations. At the same time, doctors’ low susceptibility to advertising and other promotional activities indicates that traditional marketing tools (e.g., intensive mass advertising) may be ineffective with this group. Building long-term trust through reliable information, nutritional education, and maintaining high-quality standards would be more effective. It is also worth noting that, among the surveyed doctors, there were only some differences in the assessment of motivators for purchasing decisions between women and men (Hypothesis H2 was only partially confirmed for 6 out of 26 variables). Women focus more on dairy products’ health-enhancing and nutritional aspects, while men pay more attention to practical features. To design offerings and marketing communications more effectively, food policy institutions and producers should differentiate messages and product design according to consumers’ gender.

The exploratory factor analysis conducted in our study revealed different arrangements of latent factors (i.e., implicit motivations) among the female and male decision makers (thus confirming Hypothesis H3). While both genders shared the predominant health-promoting motivation, clear structural differences emerged in the subsequent factors. This suggests that the set of characteristics that influence product choices differs between women and men. For instance, women may consider ecological, qualitative, and sensory aspects as part of a single latent factor, whereas men tend to group certain practical characteristics separately. These personalised determinants of purchasing decisions imply that targeting consumers based on gender (and other criteria) is crucial for effective marketing. In other words, promotional strategies for dairy products should be tailored to specific audience groups. Our study indicates that, overall, doctors are a demanding health-conscious group. However, within this group, women may be more receptive to messages emphasising naturalness and certified quality, while men may be more receptive to messages emphasising convenience, such as practical packaging and a long shelf life. These findings offer valuable insights for food policy institutions and dairy companies.

Among the recommendations for food policy (so-called policy levers) in the context of the trend identified in the research, whereby female doctors pay more attention to natural (organic/bio) and certified food, and both sexes share a motivation to promote health, the following can be mentioned: i) strengthening labelling and quality certification standards, ii) increasing the visibility of certification and health information on the front of the packaging (Front-of-Pack, FOP), iii) nutritional advice campaigns conducted by doctors, iv) training and education of doctors in the interpretation of labels and food safety. All of the proposed policy levers influence key elements of TPB – attitudes (e.g., through reliable information and education), subjective norms (e.g., through the authority of doctors and institutional standards), and perceived behavioural control (e.g., simplification of labels, increased legibility of certificates). As a result, they reinforce the intention to purchase certified and health-promoting products, and doctors, as a group with high authority, transfer these patterns to the wider population, reinforcing social norms of rational food choice. Increased information transparency (i) improves perceived behavioural control – consumers (doctors and their patients) may feel more confident that they can recognise a high-quality product and make the right decision. Credible certifications also reinforce positive attitudes towards products of proven quality and safety, and thus the propensity to choose them. Clear labelling (ii) acts as a normative stimulus: consumers interpret it as a socially accepted standard for healthy choices. Within the TPB, this reinforces subjective norms (‘others choose it, so I should too’) and at the same time increases perceived control by simplifying the purchasing decision. Doctors, as trusted opinion leaders, shape patients’ subjective norms – their recommendations (iii) give social approval to the choice of safe food products. These actions also reinforce positive attitudes towards healthy behaviours. Developing knowledge and competence in food safety among doctors (iv) can increase doctors’ sense of behavioural control as consumers and their ability to effectively communicate reliable information to patients. As a result, their own propensity to choose certified, i.e., healthy, products and their influence on the purchasing decisions of other consumers may increase.

Despite the limitations of a non-representative sample, the survey confirms that Polish doctors, as dairy consumers, are characterised by a high degree of rationalism and shopping conservatism. This translates into high product requirements and limited sensitivity to short-term trends or aggressive marketing. Consequently, their purchasing decisions are deliberate and consistent, presenting challenges but also opportunities for the dairy industry. Producers must meet high standards (e.g., high nutritional value, no artificial preservatives and quality confirmed by testing), but gaining the trust of such a demanding group can result in long-term loyalty. In an era of increasing competition and changing trends (e.g., the growing popularity of plant-based diets), understanding the motivations and barriers of influential consumer groups such as doctors is highly relevant. Comparing doctors’ purchasing behaviour with that of other consumer groups (e.g., people without medical training) could reveal the extent to which expert knowledge translates into everyday dietary choices. The present results suggest that education and consumer awareness significantly influence preferences in the dairy market. Health factors are important, but taste and price remain significant in consumers’ multifaceted decision-making processes.

The results of this study have a number of important implications for both theory and practice. From a theoretical perspective, the results confirm the validity of the Theory of Planned Behaviour in explaining the process of nutritional decision-making in health-oriented populations. By demonstrating that attitudes and perceived behavioural control are the strongest predictors of purchase intentions among Polish doctors, this study contributes to the increasing amount of evidence suggesting that TPB remains a robust and flexible framework for modelling consumer behaviour in the dairy market. The inclusion of gender as a segmentation variable highlights the need to extend traditional TPB models with demographic moderators, which may help capture nuances in behaviour between consumer subgroups.

From a practical point of view, the research results presented in this study contribute new insights to the discussion on factors influencing purchasing decisions for dairy products, and thus to the search for ways to potentially increase the consumption of these products, especially among health-conscious consumers. Firstly, the research was conducted among a professional group of doctors, i.e., in an environment with high social trust. The attitudes and preferences of this group of consumers, including their opinions on the nutritional, ecological and sensory value of dairy products, can influence others through direct recommendations and by setting an example through their personal choices and lifestyle. Therefore, this article’s conclusions can be used to create products and communications aimed at informed consumers and to shape educational, informational and promotional tools used in food policy. Secondly, the research addresses the underrepresentation of gender in consumer behaviour research. This enables dairy producers to design marketing communications tailored to the preferences of both sexes. Furthermore, the factor analysis and respondent clustering show different segments with different motivational profiles. The results suggest that dairy producers must differentiate their marketing strategy for each target segment.

A limitation of this study is the use of a non-random sample selection, which did not allow for the construction of a sample representative of the population of doctors in Poland, nor of the general population of buyers in Poland, thus limiting the possibility of generalising the results to a wider population. On the other hand, the study has many significant advantages that give it cognitive and practical value. A key advantage is the purposeful selection of respondents from the medical service, a professional group characterised by its expertise in health and nutrition. Their views serve as a reference point for understanding the behaviour of various consumer groups and inform the design of effective educational and marketing campaigns. Selecting respondents in proportion to the population in each province ensured regional balance and increased spatial representativeness. Using the CAPI technique enabled the survey to be efficiently implemented among a professional group with limited availability. One direction for future research could be to research the motives that influence the purchasing decisions of individual purchasers – doctors – in the dairy products market, using a representative sample from other countries (including European Union countries). Additionally, comparing the motivators of purchasing decisions among doctors with those of other groups of respondents without medical education or knowledge would be interesting.

## Supporting information

S1 Table Database_Respondents’ answers to the question: How important are these factors in your decision to purchase dairy products? (DOCX)

S2 Table Communality of variables in exploratory factor analysis. (DOCX)

S3 Table Results of exploratory factor analysis of purchase decision motives in the dairy product market (for total respondents and women and men). (DOCX)
