# Supplementary material for: Determinants of dairy products purchase decisions among polish doctors: A gender-based analysis
Source: PLoS One. 2026 Feb 27;21(2):e0339849. doi: 10.1371/journal.pone.0339849 (PMC12948115; doi:10.1371/journal.pone.0339849)
Supplement: S1 Table — (DOCX) [file pone.0339849.s001.docx]

**Determinants of dairy products purchase decisions among polish doctors: A gender-based analysis**

**Table 1. Database_Respondents' answers to the question: How important are these factors in your decision to purchase dairy products?**

| RESP_ID | Sex | Year of birth | How important are these factors in your decision to purchase dairy products? | | | | | | | | | | | | | | | | | | | | | | | | | |
| --- | --- | --- | --- | --- | --- | --- | --- | --- | --- | --- | --- | --- | --- | --- | --- | --- | --- | --- | --- | --- | --- | --- | --- | --- | --- | --- | --- | --- |
|  |  |  | q9_r1 | q9_r2 | q9_r3 | q9_r4 | q9_r5 | q9_r6 | q9_r7 | q9_r8 | q9_r9 | q9_r10 | q9_r11 | q9_r12 | q9_r13 | q9_r14 | q9_r15 | q9_r16 | q9_r17 | q9_r18 | q9_r19 | q9_r20 | q9_r21 | q9_r22 | q9_r23 | q9_r24 | q9_r25 | q9_r26 |
| 1 | 1 | 1989 | 4 | 2 | 1 | 3 | 4 | 4 | 1 | 4 | 2 | 2 | 1 | 4 | 4 | 2 | 3 | 4 | 4 | 4 | 1 | 3 | 2 | 4 | 2 | 4 | 4 | 3 |
| 9 | 1 | 1988 | 4 | 4 | 5 | 4 | 4 | 4 | 3 | 4 | 3 | 3 | 4 | 3 | 4 | 2 | 3 | 3 | 4 | 4 | 3 | 3 | 3 | 3 | 2 | 4 | 3 | 4 |
| 14 | 1 | 1990 | 3 | 5 | 3 | 3 | 5 | 3 | 3 | 3 | 3 | 3 | 1 | 3 | 5 | 3 | 5 | 5 | 5 | 5 | 5 | 5 | 5 | 3 | 1 | 5 | 5 | 5 |
| 17 | 1 | 1982 | 3 | 3 | 3 | 3 | 3 | 3 | 3 | 3 | 3 | 3 | 3 | 3 | 3 | 3 | 3 | 3 | 3 | 3 | 3 | 3 | 3 | 3 | 3 | 3 | 3 | 3 |
| 19 | 1 | 1988 | 5 | 5 | 3 | 5 | 4 | 5 | 5 | 5 | 5 | 3 | 5 | 5 | 5 | 4 | 3 | 3 | 4 | 3 | 3 | 3 | 3 | 3 | 3 | 3 | 4 | 5 |
| 24 | 1 | 1990 | 5 | 5 | 5 | 5 | 5 | 5 | 5 | 5 | 3 | 4 | 5 | 4 | 5 | 1 | 3 | 3 | 1 | 3 | 4 | 5 | 5 | 5 | 1 | 4 | 5 | 4 |
| 26 | 1 | 1992 | 5 | 5 | 4 | 5 | 4 | 5 | 5 | 5 | 5 | 2 | 5 | 5 | 3 | 1 | 3 | 1 | 2 | 3 | 2 | 1 | 3 | 3 | 1 | 1 | 3 | 2 |
| 31 | 1 | 1981 | 5 | 5 | 4 | 5 | 4 | 5 | 2 | 3 | 3 | 4 | 5 | 3 | 3 | 1 | 2 | 2 | 1 | 3 | 4 | 3 | 2 | 1 | 2 | 4 | 2 | 3 |
| 32 | 1 | 1981 | 5 | 5 | 4 | 5 | 5 | 5 | 4 | 4 | 5 | 5 | 5 | 4 | 5 | 5 | 2 | 4 | 4 | 4 | 4 | 4 | 2 | 2 | 5 | 4 | 4 | 3 |
| 33 | 1 | 1983 | 4 | 4 | 2 | 3 | 4 | 2 | 2 | 2 | 4 | 3 | 2 | 2 | 3 | 2 | 2 | 2 | 2 | 4 | 3 | 3 | 2 | 2 | 2 | 4 | 2 | 2 |
| 34 | 1 | 1989 | 4 | 4 | 4 | 4 | 4 | 4 | 2 | 4 | 3 | 3 | 3 | 3 | 3 | 2 | 3 | 3 | 3 | 3 | 3 | 3 | 2 | 3 | 2 | 4 | 3 | 4 |
| 36 | 1 | 1983 | 5 | 4 | 3 | 5 | 4 | 4 | 4 | 4 | 4 | 3 | 4 | 4 | 4 | 2 | 3 | 3 | 3 | 3 | 3 | 3 | 3 | 3 | 2 | 4 | 3 | 4 |
| 39 | 1 | 1983 | 5 | 4 | 3 | 4 | 4 | 4 | 4 | 4 | 3 | 3 | 2 | 3 | 4 | 4 | 4 | 4 | 4 | 4 | 3 | 4 | 4 | 4 | 4 | 5 | 5 | 4 |
| 43 | 1 | 1991 | 4 | 3 | 3 | 4 | 3 | 4 | 4 | 3 | 3 | 3 | 3 | 3 | 3 | 3 | 3 | 3 | 3 | 4 | 3 | 3 | 4 | 3 | 3 | 3 | 3 | 3 |
| 44 | 1 | 1985 | 5 | 5 | 3 | 5 | 4 | 5 | 4 | 4 | 4 | 3 | 4 | 4 | 4 | 4 | 3 | 3 | 4 | 3 | 3 | 3 | 4 | 4 | 3 | 4 | 4 | 4 |
| 47 | 1 | 1990 | 3 | 4 | 5 | 4 | 4 | 4 | 3 | 3 | 2 | 3 | 3 | 2 | 4 | 1 | 4 | 2 | 4 | 5 | 3 | 3 | 4 | 4 | 4 | 4 | 2 | 3 |
| 56 | 1 | 1990 | 5 | 5 | 3 | 5 | 5 | 3 | 5 | 3 | 1 | 1 | 5 | 5 | 5 | 3 | 3 | 3 | 5 | 5 | 3 | 3 | 5 | 5 | 3 | 5 | 3 | 3 |
| 57 | 1 | 1983 | 4 | 4 | 4 | 5 | 3 | 5 | 4 | 3 | 5 | 3 | 5 | 5 | 3 | 1 | 1 | 1 | 1 | 3 | 2 | 3 | 3 | 3 | 1 | 3 | 3 | 4 |
| 58 | 1 | 1983 | 5 | 5 | 5 | 5 | 4 | 5 | 5 | 5 | 4 | 3 | 3 | 4 | 5 | 2 | 1 | 4 | 2 | 2 | 4 | 3 | 3 | 2 | 1 | 5 | 5 | 3 |
| 60 | 1 | 1989 | 2 | 4 | 5 | 5 | 5 | 2 | 1 | 1 | 2 | 4 | 5 | 2 | 4 | 1 | 4 | 2 | 1 | 5 | 5 | 4 | 4 | 3 | 1 | 5 | 4 | 4 |
| 61 | 1 | 1997 | 2 | 2 | 2 | 2 | 2 | 2 | 2 | 2 | 2 | 2 | 2 | 2 | 2 | 2 | 2 | 2 | 2 | 2 | 2 | 2 | 2 | 2 | 2 | 2 | 2 | 2 |
| 64 | 1 | 1990 | 4 | 4 | 5 | 5 | 5 | 5 | 2 | 4 | 4 | 4 | 4 | 4 | 5 | 4 | 3 | 5 | 3 | 5 | 4 | 2 | 4 | 4 | 3 | 4 | 4 | 5 |
| 67 | 1 | 1992 | 4 | 5 | 4 | 5 | 3 | 4 | 4 | 4 | 5 | 3 | 4 | 4 | 4 | 2 | 2 | 2 | 4 | 4 | 4 | 2 | 2 | 2 | 3 | 4 | 4 | 3 |
| 73 | 1 | 1984 | 5 | 5 | 3 | 5 | 5 | 3 | 2 | 5 | 3 | 5 | 5 | 5 | 5 | 1 | 1 | 2 | 2 | 3 | 4 | 1 | 2 | 2 | 2 | 4 | 3 | 5 |
| 74 | 1 | 1981 | 3 | 2 | 2 | 2 | 3 | 3 | 3 | 2 | 3 | 2 | 2 | 2 | 3 | 3 | 3 | 3 | 3 | 2 | 2 | 2 | 2 | 3 | 3 | 2 | 2 | 2 |
| 78 | 1 | 1988 | 5 | 4 | 4 | 5 | 4 | 5 | 4 | 4 | 4 | 3 | 3 | 3 | 4 | 3 | 4 | 5 | 5 | 4 | 4 | 4 | 4 | 4 | 3 | 4 | 4 | 5 |
| 80 | 1 | 1983 | 5 | 5 | 5 | 5 | 5 | 5 | 4 | 5 | 5 | 3 | 4 | 3 | 5 | 3 | 3 | 3 | 3 | 5 | 4 | 4 | 5 | 5 | 3 | 3 | 3 | 5 |
| 84 | 1 | 1983 | 4 | 4 | 4 | 4 | 4 | 4 | 4 | 4 | 4 | 4 | 4 | 4 | 4 | 4 | 4 | 4 | 4 | 4 | 4 | 4 | 4 | 4 | 4 | 4 | 4 | 4 |
| 85 | 1 | 1993 | 3 | 3 | 2 | 4 | 2 | 3 | 2 | 2 | 3 | 2 | 2 | 3 | 3 | 2 | 2 | 3 | 3 | 4 | 2 | 2 | 2 | 4 | 2 | 4 | 2 | 2 |
| 87 | 1 | 1982 | 5 | 5 | 2 | 5 | 5 | 5 | 4 | 4 | 4 | 3 | 5 | 4 | 5 | 2 | 3 | 3 | 3 | 4 | 3 | 3 | 5 | 3 | 3 | 4 | 4 | 4 |
| 88 | 1 | 1984 | 5 | 4 | 5 | 5 | 4 | 5 | 2 | 5 | 2 | 2 | 4 | 4 | 3 | 2 | 2 | 3 | 4 | 4 | 2 | 2 | 4 | 4 | 2 | 4 | 3 | 4 |
| 90 | 1 | 1989 | 5 | 5 | 5 | 5 | 4 | 5 | 3 | 3 | 2 | 5 | 3 | 5 | 4 | 4 | 3 | 3 | 5 | 3 | 4 | 2 | 3 | 3 | 3 | 5 | 2 | 4 |
| 103 | 1 | 1992 | 5 | 4 | 5 | 4 | 4 | 5 | 4 | 4 | 4 | 4 | 4 | 4 | 4 | 2 | 3 | 2 | 3 | 3 | 4 | 3 | 3 | 3 | 4 | 4 | 4 | 2 |
| 104 | 1 | 1981 | 5 | 5 | 3 | 5 | 4 | 5 | 4 | 4 | 4 | 4 | 4 | 4 | 3 | 3 | 2 | 3 | 2 | 3 | 4 | 1 | 3 | 3 | 1 | 4 | 4 | 4 |
| 105 | 1 | 1985 | 5 | 5 | 4 | 5 | 4 | 5 | 5 | 5 | 5 | 4 | 5 | 5 | 4 | 5 | 3 | 4 | 5 | 4 | 4 | 4 | 4 | 4 | 4 | 4 | 4 | 4 |
| 106 | 1 | 1985 | 5 | 5 | 4 | 5 | 4 | 4 | 4 | 4 | 4 | 4 | 5 | 4 | 4 | 4 | 4 | 4 | 4 | 4 | 4 | 3 | 4 | 4 | 3 | 4 | 4 | 4 |
| 110 | 1 | 1984 | 5 | 5 | 5 | 5 | 5 | 5 | 3 | 5 | 4 | 5 | 5 | 5 | 4 | 1 | 3 | 1 | 3 | 3 | 5 | 1 | 4 | 3 | 1 | 3 | 5 | 5 |
| 120 | 1 | 1981 | 5 | 5 | 5 | 5 | 5 | 4 | 3 | 3 | 5 | 3 | 4 | 4 | 4 | 1 | 4 | 3 | 1 | 4 | 4 | 2 | 3 | 3 | 2 | 5 | 4 | 5 |
| 122 | 1 | 1983 | 5 | 5 | 4 | 5 | 5 | 5 | 5 | 5 | 4 | 4 | 4 | 4 | 4 | 5 | 3 | 4 | 3 | 4 | 4 | 4 | 4 | 4 | 3 | 3 | 5 | 3 |
| 133 | 1 | 1990 | 4 | 5 | 3 | 4 | 4 | 4 | 4 | 4 | 3 | 3 | 3 | 3 | 4 | 3 | 3 | 3 | 3 | 4 | 4 | 3 | 3 | 4 | 3 | 4 | 3 | 3 |
| 135 | 1 | 1990 | 5 | 5 | 5 | 5 | 5 | 5 | 5 | 5 | 5 | 5 | 5 | 5 | 5 | 5 | 5 | 5 | 5 | 5 | 5 | 5 | 5 | 5 | 5 | 5 | 5 | 5 |
| 138 | 1 | 1985 | 3 | 3 | 3 | 3 | 3 | 3 | 2 | 3 | 3 | 3 | 4 | 3 | 3 | 1 | 3 | 3 | 3 | 3 | 3 | 3 | 3 | 3 | 3 | 3 | 3 | 3 |
| 139 | 1 | 1987 | 4 | 3 | 3 | 4 | 4 | 4 | 3 | 3 | 4 | 4 | 5 | 4 | 4 | 4 | 3 | 4 | 5 | 3 | 4 | 4 | 4 | 3 | 1 | 4 | 3 | 4 |
| 140 | 1 | 1984 | 5 | 4 | 4 | 4 | 4 | 4 | 3 | 3 | 4 | 4 | 5 | 5 | 4 | 3 | 4 | 4 | 5 | 3 | 4 | 4 | 4 | 3 | 3 | 4 | 3 | 4 |
| 142 | 1 | 1987 | 5 | 5 | 3 | 5 | 5 | 5 | 5 | 5 | 5 | 3 | 3 | 4 | 3 | 1 | 1 | 1 | 1 | 2 | 3 | 1 | 3 | 1 | 1 | 4 | 3 | 3 |
| 144 | 1 | 1983 | 5 | 5 | 4 | 5 | 3 | 5 | 4 | 3 | 4 | 4 | 4 | 3 | 3 | 2 | 3 | 2 | 5 | 4 | 3 | 1 | 3 | 3 | 2 | 4 | 4 | 5 |
| 146 | 1 | 1981 | 5 | 5 | 3 | 5 | 5 | 5 | 4 | 4 | 5 | 3 | 5 | 3 | 5 | 3 | 4 | 3 | 5 | 5 | 3 | 2 | 5 | 5 | 3 | 4 | 4 | 5 |
| 149 | 1 | 1981 | 5 | 5 | 5 | 5 | 4 | 5 | 4 | 5 | 5 | 4 | 4 | 5 | 5 | 4 | 4 | 4 | 4 | 5 | 4 | 3 | 4 | 5 | 5 | 4 | 4 | 5 |
| 153 | 1 | 1984 | 5 | 5 | 3 | 5 | 3 | 5 | 5 | 5 | 4 | 5 | 4 | 5 | 5 | 1 | 1 | 2 | 2 | 2 | 1 | 4 | 2 | 1 | 1 | 5 | 3 | 4 |
| 157 | 1 | 1985 | 4 | 3 | 4 | 4 | 4 | 4 | 3 | 4 | 4 | 3 | 3 | 3 | 4 | 1 | 3 | 3 | 2 | 3 | 3 | 2 | 3 | 4 | 1 | 4 | 3 | 4 |
| 159 | 1 | 1994 | 5 | 5 | 5 | 5 | 5 | 5 | 5 | 5 | 5 | 5 | 5 | 5 | 5 | 5 | 5 | 5 | 5 | 5 | 5 | 5 | 5 | 5 | 5 | 5 | 5 | 5 |
| 165 | 1 | 1982 | 4 | 4 | 4 | 4 | 4 | 4 | 4 | 4 | 4 | 3 | 4 | 4 | 4 | 3 | 3 | 4 | 3 | 4 | 3 | 4 | 4 | 3 | 3 | 4 | 3 | 4 |
| 173 | 1 | 1988 | 5 | 5 | 3 | 5 | 5 | 5 | 5 | 5 | 4 | 3 | 5 | 4 | 4 | 2 | 2 | 2 | 2 | 3 | 2 | 2 | 4 | 2 | 2 | 4 | 4 | 2 |
| 174 | 1 | 1990 | 3 | 3 | 4 | 3 | 4 | 3 | 4 | 3 | 4 | 3 | 4 | 3 | 3 | 4 | 3 | 4 | 4 | 3 | 3 | 4 | 3 | 4 | 3 | 4 | 3 | 4 |
| 178 | 1 | 1983 | 5 | 5 | 4 | 5 | 4 | 5 | 5 | 5 | 5 | 3 | 4 | 4 | 4 | 4 | 4 | 3 | 4 | 4 | 3 | 3 | 4 | 4 | 4 | 4 | 5 | 4 |
| 186 | 1 | 1983 | 3 | 4 | 3 | 3 | 3 | 3 | 3 | 3 | 3 | 3 | 2 | 3 | 4 | 4 | 4 | 4 | 3 | 3 | 4 | 3 | 2 | 3 | 3 | 4 | 2 | 2 |
| 189 | 1 | 1991 | 4 | 4 | 2 | 2 | 2 | 3 | 2 | 4 | 4 | 4 | 2 | 4 | 2 | 2 | 2 | 2 | 3 | 4 | 2 | 2 | 3 | 2 | 4 | 2 | 4 | 4 |
| 193 | 1 | 1985 | 5 | 5 | 5 | 5 | 5 | 5 | 4 | 5 | 1 | 5 | 5 | 5 | 5 | 1 | 1 | 3 | 2 | 5 | 3 | 3 | 5 | 5 | 1 | 3 | 3 | 4 |
| 199 | 1 | 1988 | 4 | 5 | 1 | 5 | 2 | 5 | 5 | 5 | 5 | 4 | 4 | 4 | 4 | 1 | 1 | 2 | 2 | 2 | 5 | 1 | 2 | 3 | 1 | 3 | 3 | 2 |
| 2 | 1 | 1970 | 5 | 4 | 5 | 3 | 5 | 4 | 5 | 5 | 5 | 3 | 5 | 4 | 3 | 3 | 4 | 3 | 4 | 3 | 5 | 3 | 4 | 3 | 2 | 3 | 3 | 3 |
| 4 | 1 | 1963 | 2 | 2 | 3 | 2 | 2 | 2 | 4 | 3 | 3 | 3 | 4 | 2 | 4 | 3 | 3 | 2 | 3 | 2 | 4 | 2 | 3 | 2 | 2 | 4 | 2 | 4 |
| 7 | 1 | 1977 | 4 | 5 | 5 | 5 | 5 | 4 | 3 | 4 | 4 | 3 | 4 | 4 | 4 | 1 | 3 | 3 | 3 | 4 | 3 | 3 | 5 | 5 | 2 | 5 | 5 | 5 |
| 8 | 1 | 1977 | 4 | 4 | 4 | 4 | 5 | 4 | 3 | 4 | 4 | 4 | 3 | 4 | 5 | 3 | 4 | 4 | 5 | 4 | 4 | 4 | 4 | 4 | 5 | 5 | 5 | 5 |
| 10 | 1 | 1969 | 5 | 5 | 4 | 5 | 5 | 5 | 4 | 5 | 5 | 5 | 5 | 4 | 4 | 3 | 2 | 5 | 5 | 3 | 5 | 4 | 5 | 2 | 3 | 5 | 3 | 5 |
| 20 | 1 | 1967 | 4 | 3 | 5 | 3 | 5 | 4 | 2 | 4 | 3 | 3 | 4 | 2 | 4 | 1 | 3 | 1 | 4 | 4 | 3 | 2 | 4 | 3 | 2 | 4 | 4 | 4 |
| 22 | 1 | 1974 | 5 | 5 | 5 | 5 | 5 | 5 | 5 | 5 | 5 | 5 | 5 | 5 | 5 | 5 | 5 | 5 | 5 | 5 | 5 | 5 | 4 | 5 | 5 | 5 | 5 | 5 |
| 27 | 1 | 1970 | 5 | 3 | 5 | 3 | 4 | 5 | 4 | 3 | 4 | 3 | 2 | 4 | 2 | 2 | 4 | 2 | 2 | 4 | 3 | 2 | 2 | 2 | 1 | 5 | 2 | 4 |
| 28 | 1 | 1970 | 4 | 4 | 4 | 4 | 5 | 4 | 2 | 4 | 4 | 4 | 4 | 4 | 1 | 1 | 3 | 3 | 1 | 3 | 4 | 2 | 4 | 1 | 1 | 5 | 4 | 5 |
| 30 | 1 | 1961 | 5 | 3 | 4 | 5 | 4 | 5 | 5 | 5 | 4 | 4 | 1 | 4 | 5 | 1 | 1 | 3 | 3 | 4 | 4 | 3 | 4 | 5 | 1 | 5 | 2 | 5 |
| 35 | 1 | 1974 | 5 | 5 | 5 | 5 | 5 | 5 | 3 | 5 | 3 | 4 | 5 | 3 | 5 | 2 | 3 | 1 | 1 | 5 | 2 | 1 | 5 | 5 | 1 | 5 | 3 | 5 |
| 42 | 1 | 1968 | 5 | 5 | 5 | 5 | 5 | 4 | 5 | 5 | 5 | 4 | 5 | 5 | 5 | 5 | 5 | 5 | 5 | 5 | 4 | 5 | 5 | 4 | 5 | 5 | 5 | 4 |
| 46 | 1 | 1974 | 3 | 3 | 3 | 4 | 4 | 4 | 3 | 3 | 4 | 4 | 4 | 4 | 3 | 4 | 4 | 4 | 2 | 4 | 4 | 3 | 4 | 4 | 4 | 3 | 4 | 3 |
| 53 | 1 | 1960 | 5 | 5 | 5 | 5 | 5 | 5 | 5 | 5 | 5 | 5 | 5 | 5 | 5 | 5 | 3 | 4 | 5 | 5 | 5 | 4 | 4 | 3 | 3 | 4 | 5 | 4 |
| 54 | 1 | 1969 | 4 | 4 | 5 | 4 | 5 | 4 | 4 | 5 | 4 | 4 | 4 | 4 | 4 | 4 | 2 | 4 | 4 | 3 | 5 | 4 | 4 | 3 | 3 | 4 | 4 | 4 |
| 59 | 1 | 1976 | 4 | 4 | 4 | 4 | 4 | 4 | 2 | 4 | 4 | 4 | 4 | 1 | 4 | 1 | 4 | 3 | 4 | 5 | 4 | 3 | 4 | 4 | 1 | 4 | 3 | 4 |
| 62 | 1 | 1968 | 5 | 5 | 5 | 5 | 5 | 5 | 5 | 5 | 4 | 5 | 5 | 5 | 5 | 5 | 4 | 4 | 4 | 5 | 5 | 4 | 4 | 4 | 5 | 5 | 5 | 5 |
| 63 | 1 | 1973 | 4 | 4 | 3 | 5 | 5 | 4 | 4 | 5 | 4 | 4 | 4 | 5 | 4 | 5 | 3 | 3 | 3 | 4 | 4 | 4 | 3 | 4 | 3 | 5 | 4 | 5 |
| 68 | 1 | 1968 | 4 | 5 | 5 | 5 | 4 | 5 | 3 | 4 | 4 | 4 | 4 | 4 | 4 | 4 | 3 | 3 | 3 | 3 | 4 | 3 | 3 | 3 | 3 | 3 | 3 | 4 |
| 71 | 1 | 1972 | 5 | 5 | 4 | 5 | 5 | 4 | 5 | 4 | 3 | 3 | 5 | 5 | 4 | 3 | 4 | 5 | 3 | 4 | 3 | 4 | 3 | 4 | 4 | 3 | 4 | 4 |
| 77 | 1 | 1968 | 5 | 5 | 5 | 5 | 5 | 5 | 5 | 5 | 5 | 5 | 4 | 5 | 5 | 5 | 5 | 4 | 5 | 5 | 5 | 5 | 5 | 5 | 5 | 5 | 5 | 5 |
| 82 | 1 | 1969 | 4 | 4 | 5 | 4 | 4 | 4 | 3 | 4 | 3 | 4 | 3 | 3 | 4 | 4 | 3 | 4 | 3 | 5 | 3 | 4 | 3 | 4 | 3 | 4 | 3 | 4 |
| 86 | 1 | 1974 | 5 | 5 | 4 | 5 | 5 | 5 | 3 | 3 | 4 | 4 | 4 | 5 | 5 | 3 | 2 | 2 | 4 | 3 | 4 | 3 | 4 | 3 | 1 | 4 | 4 | 5 |
| 89 | 1 | 1976 | 4 | 4 | 4 | 5 | 4 | 4 | 4 | 4 | 3 | 4 | 5 | 2 | 4 | 3 | 2 | 4 | 5 | 4 | 4 | 2 | 4 | 4 | 2 | 4 | 2 | 3 |
| 92 | 1 | 1965 | 2 | 5 | 1 | 5 | 3 | 3 | 2 | 2 | 1 | 1 | 1 | 1 | 2 | 1 | 1 | 1 | 1 | 2 | 2 | 1 | 2 | 2 | 1 | 2 | 1 | 1 |
| 93 | 1 | 1965 | 4 | 4 | 3 | 4 | 4 | 4 | 4 | 4 | 4 | 4 | 3 | 4 | 4 | 2 | 2 | 2 | 2 | 4 | 4 | 4 | 3 | 4 | 3 | 3 | 4 | 4 |
| 95 | 1 | 1979 | 5 | 5 | 3 | 5 | 3 | 5 | 4 | 4 | 4 | 4 | 3 | 4 | 3 | 3 | 1 | 1 | 2 | 3 | 3 | 3 | 3 | 3 | 1 | 3 | 3 | 4 |
| 96 | 1 | 1949 | 4 | 5 | 5 | 4 | 4 | 5 | 4 | 5 | 4 | 4 | 5 | 5 | 4 | 4 | 4 | 3 | 4 | 4 | 5 | 4 | 4 | 4 | 5 | 4 | 4 | 4 |
| 100 | 1 | 1973 | 5 | 4 | 3 | 5 | 3 | 4 | 3 | 3 | 5 | 4 | 4 | 4 | 3 | 2 | 3 | 3 | 3 | 3 | 4 | 3 | 3 | 4 | 4 | 3 | 4 | 2 |
| 102 | 1 | 1975 | 5 | 5 | 5 | 4 | 5 | 5 | 4 | 4 | 4 | 5 | 4 | 4 | 5 | 2 | 4 | 5 | 5 | 4 | 5 | 2 | 5 | 4 | 3 | 4 | 5 | 5 |
| 107 | 1 | 1964 | 5 | 4 | 4 | 5 | 5 | 5 | 4 | 5 | 4 | 5 | 5 | 4 | 5 | 4 | 4 | 4 | 4 | 4 | 4 | 4 | 5 | 4 | 3 | 5 | 5 | 4 |
| 112 | 1 | 1963 | 5 | 4 | 4 | 5 | 5 | 4 | 3 | 4 | 5 | 4 | 3 | 4 | 5 | 4 | 2 | 4 | 3 | 4 | 4 | 3 | 4 | 3 | 3 | 5 | 5 | 4 |
| 113 | 1 | 1977 | 3 | 3 | 5 | 2 | 3 | 3 | 2 | 2 | 3 | 2 | 2 | 2 | 3 | 2 | 2 | 3 | 2 | 3 | 3 | 3 | 2 | 2 | 3 | 3 | 2 | 2 |
| 114 | 1 | 1965 | 4 | 5 | 5 | 5 | 5 | 5 | 3 | 5 | 4 | 4 | 5 | 5 | 4 | 3 | 3 | 3 | 3 | 5 | 4 | 4 | 4 | 5 | 3 | 3 | 3 | 4 |
| 115 | 1 | 1975 | 5 | 5 | 5 | 5 | 5 | 5 | 5 | 5 | 4 | 5 | 5 | 5 | 5 | 5 | 3 | 5 | 5 | 5 | 4 | 4 | 3 | 5 | 4 | 4 | 5 | 5 |
| 116 | 1 | 1963 | 4 | 3 | 4 | 4 | 3 | 2 | 2 | 2 | 2 | 2 | 2 | 2 | 2 | 1 | 1 | 1 | 2 | 4 | 2 | 4 | 1 | 3 | 1 | 4 | 3 | 4 |
| 118 | 1 | 1979 | 1 | 3 | 1 | 5 | 1 | 5 | 4 | 5 | 1 | 1 | 3 | 4 | 1 | 1 | 1 | 1 | 1 | 1 | 1 | 1 | 1 | 1 | 1 | 1 | 1 | 1 |
| 119 | 1 | 1978 | 4 | 4 | 4 | 4 | 5 | 4 | 4 | 5 | 4 | 3 | 4 | 4 | 4 | 4 | 4 | 4 | 3 | 4 | 3 | 4 | 4 | 4 | 4 | 4 | 4 | 4 |
| 121 | 1 | 1974 | 5 | 5 | 5 | 5 | 4 | 5 | 4 | 5 | 3 | 4 | 3 | 3 | 5 | 2 | 1 | 2 | 2 | 5 | 4 | 2 | 4 | 4 | 2 | 1 | 2 | 5 |
| 127 | 1 | 1967 | 4 | 3 | 4 | 5 | 5 | 5 | 1 | 3 | 5 | 3 | 3 | 4 | 5 | 1 | 1 | 4 | 1 | 5 | 1 | 1 | 3 | 5 | 1 | 3 | 4 | 2 |
| 128 | 1 | 1967 | 4 | 4 | 4 | 4 | 4 | 5 | 3 | 3 | 4 | 3 | 4 | 4 | 4 | 2 | 3 | 3 | 3 | 4 | 3 | 3 | 3 | 3 | 3 | 4 | 3 | 3 |
| 131 | 1 | 1976 | 5 | 5 | 4 | 5 | 5 | 5 | 5 | 5 | 4 | 3 | 5 | 5 | 5 | 4 | 2 | 2 | 2 | 4 | 3 | 2 | 4 | 4 | 2 | 5 | 2 | 4 |
| 134 | 1 | 1958 | 5 | 5 | 5 | 5 | 5 | 5 | 5 | 5 | 4 | 5 | 4 | 4 | 5 | 2 | 3 | 3 | 4 | 3 | 5 | 3 | 4 | 3 | 3 | 4 | 4 | 4 |
| 136 | 1 | 1964 | 3 | 5 | 5 | 5 | 3 | 5 | 3 | 1 | 5 | 2 | 1 | 5 | 5 | 1 | 1 | 1 | 1 | 1 | 3 | 1 | 2 | 5 | 1 | 5 | 4 | 4 |
| 137 | 1 | 1965 | 3 | 3 | 4 | 4 | 3 | 1 | 1 | 3 | 2 | 3 | 3 | 4 | 2 | 1 | 1 | 1 | 1 | 1 | 3 | 1 | 1 | 1 | 1 | 2 | 2 | 3 |
| 145 | 1 | 1966 | 5 | 5 | 4 | 5 | 4 | 5 | 5 | 4 | 5 | 4 | 5 | 4 | 3 | 2 | 3 | 3 | 3 | 4 | 4 | 4 | 3 | 4 | 3 | 3 | 3 | 4 |
| 148 | 1 | 1940 | 5 | 5 | 5 | 4 | 5 | 4 | 4 | 4 | 3 | 4 | 3 | 4 | 4 | 3 | 2 | 4 | 2 | 5 | 4 | 4 | 4 | 5 | 4 | 4 | 4 | 4 |
| 150 | 1 | 1979 | 5 | 4 | 5 | 5 | 4 | 5 | 4 | 5 | 4 | 4 | 4 | 4 | 4 | 3 | 4 | 3 | 3 | 4 | 5 | 3 | 3 | 3 | 4 | 4 | 4 | 4 |
| 154 | 1 | 1954 | 5 | 5 | 5 | 5 | 5 | 5 | 5 | 5 | 5 | 4 | 5 | 4 | 4 | 4 | 2 | 4 | 4 | 4 | 4 | 4 | 5 | 5 | 2 | 5 | 5 | 3 |
| 155 | 1 | 1968 | 4 | 2 | 3 | 3 | 3 | 3 | 3 | 3 | 4 | 3 | 3 | 4 | 4 | 4 | 3 | 4 | 4 | 4 | 3 | 4 | 3 | 4 | 3 | 4 | 4 | 4 |
| 161 | 1 | 1974 | 4 | 4 | 4 | 3 | 4 | 3 | 4 | 3 | 3 | 4 | 4 | 4 | 4 | 4 | 3 | 3 | 3 | 4 | 4 | 3 | 3 | 4 | 4 | 4 | 3 | 4 |
| 164 | 1 | 1973 | 4 | 4 | 5 | 4 | 5 | 5 | 3 | 4 | 3 | 2 | 3 | 2 | 4 | 4 | 2 | 3 | 3 | 3 | 2 | 2 | 3 | 3 | 1 | 3 | 2 | 5 |
| 179 | 1 | 1972 | 4 | 4 | 3 | 4 | 4 | 2 | 2 | 4 | 2 | 4 | 2 | 3 | 3 | 1 | 2 | 3 | 3 | 4 | 4 | 4 | 4 | 3 | 2 | 5 | 2 | 4 |
| 180 | 1 | 1973 | 5 | 5 | 4 | 5 | 5 | 5 | 4 | 5 | 5 | 5 | 5 | 5 | 4 | 5 | 5 | 4 | 5 | 5 | 5 | 5 | 5 | 5 | 4 | 5 | 5 | 5 |
| 181 | 1 | 1960 | 5 | 5 | 5 | 5 | 5 | 5 | 5 | 5 | 4 | 4 | 2 | 4 | 5 | 3 | 1 | 2 | 1 | 4 | 4 | 2 | 2 | 3 | 1 | 2 | 3 | 4 |
| 182 | 1 | 1959 | 2 | 3 | 4 | 5 | 4 | 4 | 3 | 4 | 4 | 4 | 3 | 5 | 4 | 3 | 3 | 3 | 2 | 5 | 4 | 4 | 4 | 3 | 1 | 4 | 5 | 3 |
| 185 | 1 | 1962 | 3 | 3 | 1 | 1 | 1 | 1 | 1 | 1 | 1 | 3 | 1 | 1 | 5 | 1 | 3 | 1 | 1 | 3 | 1 | 1 | 5 | 1 | 1 | 5 | 1 | 5 |
| 188 | 1 | 1973 | 4 | 4 | 5 | 4 | 5 | 5 | 4 | 3 | 3 | 4 | 3 | 3 | 5 | 3 | 3 | 4 | 3 | 4 | 4 | 4 | 5 | 5 | 3 | 4 | 3 | 4 |
| 194 | 1 | 1967 | 5 | 5 | 5 | 5 | 5 | 4 | 1 | 3 | 4 | 4 | 4 | 4 | 4 | 3 | 3 | 3 | 3 | 3 | 4 | 4 | 3 | 3 | 3 | 4 | 4 | 4 |
| 196 | 1 | 1970 | 5 | 5 | 5 | 5 | 5 | 5 | 4 | 4 | 4 | 5 | 3 | 5 | 5 | 3 | 3 | 4 | 3 | 5 | 5 | 4 | 5 | 5 | 3 | 5 | 4 | 5 |
| 200 | 1 | 1954 | 5 | 4 | 3 | 4 | 5 | 5 | 4 | 3 | 4 | 3 | 4 | 5 | 3 | 4 | 3 | 3 | 3 | 1 | 3 | 2 | 1 | 3 | 3 | 5 | 3 | 3 |
| 3 | 2 | 1991 | 5 | 5 | 5 | 5 | 4 | 5 | 2 | 5 | 4 | 4 | 5 | 4 | 5 | 2 | 4 | 3 | 4 | 5 | 4 | 5 | 5 | 1 | 1 | 2 | 4 | 5 |
| 12 | 2 | 1980 | 4 | 4 | 3 | 3 | 4 | 2 | 2 | 3 | 2 | 2 | 2 | 2 | 4 | 3 | 1 | 3 | 2 | 2 | 3 | 3 | 3 | 3 | 2 | 5 | 4 | 3 |
| 13 | 2 | 1989 | 4 | 4 | 4 | 5 | 4 | 4 | 5 | 4 | 4 | 2 | 2 | 5 | 4 | 3 | 5 | 2 | 3 | 3 | 4 | 4 | 4 | 3 | 2 | 4 | 3 | 3 |
| 15 | 2 | 1992 | 3 | 3 | 3 | 3 | 3 | 3 | 3 | 3 | 3 | 3 | 3 | 3 | 3 | 3 | 3 | 3 | 3 | 3 | 3 | 3 | 3 | 3 | 3 | 3 | 3 | 3 |
| 16 | 2 | 1994 | 3 | 3 | 3 | 3 | 3 | 3 | 3 | 3 | 3 | 3 | 3 | 3 | 3 | 3 | 3 | 3 | 3 | 3 | 3 | 3 | 3 | 3 | 3 | 3 | 3 | 3 |
| 18 | 2 | 1988 | 4 | 5 | 5 | 5 | 4 | 5 | 1 | 1 | 3 | 2 | 5 | 4 | 4 | 1 | 1 | 3 | 3 | 3 | 4 | 2 | 5 | 1 | 3 | 4 | 4 | 4 |
| 21 | 2 | 1992 | 3 | 4 | 4 | 5 | 5 | 5 | 1 | 3 | 4 | 3 | 4 | 4 | 4 | 2 | 4 | 4 | 4 | 4 | 3 | 5 | 4 | 4 | 2 | 5 | 4 | 5 |
| 25 | 2 | 1986 | 5 | 3 | 4 | 5 | 4 | 5 | 4 | 3 | 4 | 2 | 2 | 2 | 5 | 4 | 4 | 3 | 4 | 3 | 2 | 2 | 4 | 5 | 3 | 4 | 4 | 3 |
| 29 | 2 | 1983 | 4 | 4 | 5 | 5 | 5 | 5 | 2 | 3 | 3 | 2 | 3 | 3 | 4 | 3 | 1 | 3 | 2 | 4 | 3 | 2 | 4 | 4 | 2 | 4 | 3 | 4 |
| 38 | 2 | 1980 | 5 | 5 | 4 | 5 | 4 | 5 | 4 | 3 | 4 | 4 | 5 | 4 | 4 | 2 | 3 | 4 | 3 | 4 | 3 | 2 | 4 | 3 | 2 | 2 | 2 | 3 |
| 45 | 2 | 1987 | 3 | 3 | 5 | 4 | 4 | 3 | 3 | 4 | 3 | 3 | 5 | 4 | 5 | 1 | 3 | 2 | 4 | 2 | 3 | 2 | 3 | 4 | 1 | 5 | 2 | 5 |
| 48 | 2 | 1993 | 3 | 3 | 5 | 4 | 1 | 2 | 1 | 1 | 1 | 1 | 4 | 1 | 5 | 1 | 1 | 1 | 5 | 5 | 1 | 1 | 4 | 5 | 1 | 3 | 1 | 4 |
| 49 | 2 | 1987 | 4 | 5 | 5 | 5 | 4 | 5 | 4 | 1 | 3 | 3 | 4 | 5 | 3 | 1 | 1 | 1 | 4 | 5 | 1 | 1 | 4 | 1 | 1 | 1 | 3 | 1 |
| 51 | 2 | 1992 | 5 | 4 | 3 | 4 | 5 | 5 | 3 | 4 | 4 | 3 | 3 | 4 | 4 | 2 | 4 | 2 | 5 | 4 | 3 | 3 | 3 | 5 | 3 | 4 | 4 | 4 |
| 52 | 2 | 1993 | 5 | 5 | 3 | 5 | 4 | 4 | 4 | 3 | 3 | 3 | 3 | 1 | 4 | 1 | 1 | 3 | 1 | 4 | 3 | 3 | 5 | 3 | 1 | 4 | 1 | 1 |
| 65 | 2 | 1994 | 3 | 3 | 4 | 3 | 5 | 5 | 3 | 3 | 4 | 3 | 2 | 5 | 4 | 4 | 3 | 5 | 5 | 3 | 5 | 3 | 3 | 3 | 2 | 5 | 5 | 4 |
| 66 | 2 | 1982 | 4 | 4 | 4 | 4 | 4 | 5 | 3 | 3 | 3 | 4 | 4 | 4 | 4 | 4 | 3 | 3 | 4 | 3 | 4 | 3 | 4 | 4 | 3 | 4 | 4 | 4 |
| 69 | 2 | 1997 | 5 | 4 | 4 | 4 | 4 | 4 | 4 | 5 | 3 | 3 | 4 | 4 | 4 | 1 | 2 | 4 | 3 | 5 | 3 | 4 | 3 | 3 | 1 | 4 | 3 | 4 |
| 75 | 2 | 1991 | 5 | 5 | 4 | 5 | 5 | 5 | 5 | 5 | 5 | 5 | 5 | 4 | 5 | 5 | 2 | 5 | 2 | 3 | 2 | 1 | 5 | 5 | 2 | 4 | 5 | 5 |
| 76 | 2 | 1991 | 4 | 5 | 3 | 4 | 5 | 4 | 4 | 4 | 3 | 2 | 5 | 3 | 3 | 4 | 3 | 3 | 3 | 4 | 3 | 4 | 3 | 3 | 3 | 3 | 4 | 4 |
| 91 | 2 | 1980 | 4 | 4 | 5 | 3 | 5 | 4 | 2 | 1 | 3 | 2 | 2 | 2 | 4 | 4 | 1 | 3 | 4 | 1 | 4 | 3 | 4 | 3 | 4 | 4 | 5 | 5 |
| 97 | 2 | 1996 | 4 | 5 | 5 | 4 | 4 | 4 | 4 | 4 | 4 | 4 | 5 | 4 | 5 | 3 | 3 | 4 | 4 | 5 | 3 | 3 | 4 | 4 | 2 | 4 | 4 | 4 |
| 99 | 2 | 1984 | 4 | 4 | 5 | 5 | 4 | 3 | 3 | 4 | 4 | 2 | 2 | 3 | 4 | 1 | 2 | 1 | 4 | 4 | 3 | 1 | 4 | 2 | 1 | 4 | 2 | 4 |
| 108 | 2 | 1980 | 5 | 5 | 4 | 5 | 5 | 5 | 5 | 5 | 5 | 4 | 4 | 4 | 5 | 4 | 3 | 3 | 4 | 4 | 4 | 4 | 3 | 5 | 2 | 4 | 4 | 4 |
| 129 | 2 | 1992 | 5 | 3 | 4 | 5 | 5 | 5 | 2 | 4 | 4 | 4 | 3 | 4 | 5 | 5 | 3 | 3 | 5 | 5 | 3 | 2 | 5 | 4 | 1 | 4 | 4 | 3 |
| 143 | 2 | 1989 | 3 | 2 | 2 | 2 | 3 | 3 | 2 | 2 | 2 | 3 | 2 | 2 | 2 | 2 | 2 | 1 | 2 | 3 | 3 | 4 | 1 | 1 | 2 | 1 | 3 | 2 |
| 147 | 2 | 1983 | 5 | 5 | 4 | 5 | 5 | 4 | 4 | 4 | 5 | 5 | 4 | 5 | 4 | 1 | 4 | 4 | 4 | 4 | 4 | 4 | 5 | 5 | 1 | 5 | 4 | 5 |
| 158 | 2 | 1983 | 5 | 4 | 5 | 4 | 3 | 5 | 4 | 4 | 5 | 3 | 4 | 4 | 3 | 2 | 2 | 4 | 2 | 3 | 3 | 3 | 3 | 3 | 1 | 3 | 2 | 3 |
| 160 | 2 | 1986 | 4 | 4 | 5 | 5 | 5 | 3 | 2 | 4 | 4 | 3 | 5 | 5 | 5 | 1 | 3 | 5 | 4 | 5 | 3 | 5 | 4 | 3 | 4 | 3 | 5 | 4 |
| 163 | 2 | 1997 | 5 | 5 | 2 | 5 | 3 | 4 | 1 | 3 | 3 | 3 | 3 | 4 | 1 | 1 | 1 | 1 | 1 | 3 | 2 | 1 | 1 | 2 | 1 | 4 | 1 | 2 |
| 166 | 2 | 1980 | 4 | 4 | 4 | 2 | 3 | 4 | 4 | 3 | 4 | 1 | 1 | 4 | 5 | 1 | 3 | 1 | 4 | 1 | 1 | 2 | 5 | 1 | 1 | 4 | 2 | 4 |
| 168 | 2 | 1981 | 4 | 4 | 5 | 3 | 5 | 3 | 2 | 4 | 2 | 4 | 3 | 3 | 5 | 1 | 2 | 5 | 4 | 4 | 4 | 4 | 4 | 5 | 4 | 4 | 5 | 4 |
| 171 | 2 | 1986 | 3 | 3 | 4 | 2 | 2 | 2 | 2 | 3 | 2 | 2 | 5 | 2 | 3 | 2 | 2 | 3 | 2 | 4 | 2 | 4 | 4 | 2 | 3 | 4 | 4 | 5 |
| 176 | 2 | 1982 | 3 | 5 | 5 | 4 | 4 | 4 | 3 | 3 | 3 | 4 | 4 | 4 | 4 | 2 | 4 | 3 | 2 | 3 | 3 | 3 | 3 | 2 | 1 | 4 | 2 | 4 |
| 5 | 2 | 1963 | 2 | 4 | 4 | 2 | 3 | 4 | 2 | 4 | 3 | 3 | 4 | 4 | 3 | 4 | 3 | 3 | 3 | 3 | 4 | 2 | 4 | 3 | 3 | 4 | 3 | 3 |
| 6 | 2 | 1971 | 4 | 3 | 5 | 4 | 5 | 4 | 3 | 4 | 4 | 4 | 2 | 4 | 3 | 2 | 2 | 3 | 3 | 5 | 3 | 4 | 3 | 3 | 5 | 5 | 4 | 2 |
| 11 | 2 | 1962 | 3 | 5 | 5 | 4 | 5 | 5 | 2 | 5 | 3 | 3 | 4 | 2 | 5 | 1 | 4 | 2 | 2 | 2 | 5 | 3 | 3 | 2 | 5 | 5 | 3 | 4 |
| 23 | 2 | 1966 | 5 | 5 | 4 | 5 | 5 | 5 | 4 | 4 | 5 | 5 | 5 | 5 | 5 | 5 | 4 | 3 | 4 | 5 | 5 | 4 | 4 | 4 | 3 | 5 | 4 | 5 |
| 37 | 2 | 1966 | 3 | 3 | 4 | 4 | 3 | 4 | 1 | 3 | 2 | 3 | 4 | 3 | 3 | 2 | 2 | 2 | 2 | 2 | 3 | 3 | 3 | 1 | 2 | 3 | 2 | 4 |
| 40 | 2 | 1971 | 2 | 3 | 5 | 2 | 5 | 3 | 3 | 2 | 3 | 4 | 4 | 4 | 4 | 2 | 2 | 4 | 4 | 4 | 4 | 4 | 4 | 5 | 4 | 4 | 4 | 4 |
| 41 | 2 | 1948 | 5 | 5 | 3 | 5 | 4 | 5 | 4 | 4 | 5 | 5 | 2 | 4 | 5 | 1 | 2 | 1 | 2 | 1 | 4 | 2 | 4 | 1 | 1 | 3 | 4 | 5 |
| 50 | 2 | 1969 | 5 | 5 | 5 | 5 | 5 | 4 | 4 | 5 | 4 | 4 | 4 | 4 | 4 | 3 | 3 | 3 | 4 | 5 | 5 | 4 | 5 | 4 | 4 | 4 | 4 | 4 |
| 55 | 2 | 1974 | 5 | 5 | 5 | 5 | 4 | 5 | 2 | 4 | 2 | 4 | 5 | 4 | 4 | 2 | 2 | 3 | 2 | 3 | 4 | 3 | 4 | 5 | 1 | 3 | 1 | 4 |
| 70 | 2 | 1957 | 5 | 5 | 5 | 5 | 5 | 5 | 5 | 5 | 5 | 5 | 5 | 5 | 5 | 5 | 5 | 5 | 5 | 5 | 5 | 5 | 5 | 5 | 5 | 5 | 5 | 5 |
| 72 | 2 | 1968 | 3 | 2 | 5 | 5 | 5 | 5 | 3 | 4 | 5 | 5 | 5 | 5 | 5 | 4 | 4 | 5 | 4 | 5 | 5 | 3 | 3 | 5 | 2 | 5 | 5 | 4 |
| 79 | 2 | 1962 | 5 | 5 | 5 | 5 | 4 | 5 | 4 | 5 | 5 | 5 | 5 | 4 | 5 | 3 | 4 | 3 | 4 | 5 | 5 | 4 | 5 | 5 | 3 | 5 | 4 | 5 |
| 81 | 2 | 1968 | 4 | 3 | 3 | 3 | 3 | 3 | 2 | 3 | 3 | 3 | 3 | 3 | 3 | 1 | 2 | 3 | 3 | 3 | 3 | 3 | 4 | 3 | 3 | 4 | 3 | 3 |
| 83 | 2 | 1971 | 3 | 4 | 5 | 4 | 5 | 3 | 2 | 3 | 3 | 4 | 4 | 3 | 4 | 2 | 2 | 2 | 2 | 3 | 4 | 2 | 4 | 2 | 2 | 5 | 2 | 3 |
| 94 | 2 | 1969 | 4 | 4 | 4 | 5 | 5 | 5 | 3 | 5 | 5 | 4 | 4 | 5 | 4 | 2 | 3 | 4 | 3 | 4 | 4 | 3 | 4 | 4 | 3 | 4 | 4 | 3 |
| 98 | 2 | 1979 | 5 | 4 | 2 | 5 | 4 | 4 | 4 | 5 | 4 | 4 | 4 | 4 | 3 | 2 | 3 | 4 | 3 | 4 | 4 | 3 | 4 | 4 | 3 | 4 | 3 | 4 |
| 101 | 2 | 1977 | 3 | 3 | 3 | 3 | 4 | 3 | 2 | 3 | 3 | 3 | 3 | 3 | 3 | 3 | 4 | 4 | 3 | 5 | 4 | 4 | 3 | 4 | 4 | 3 | 3 | 4 |
| 109 | 2 | 1979 | 4 | 5 | 2 | 5 | 5 | 5 | 4 | 4 | 4 | 2 | 5 | 4 | 4 | 4 | 4 | 2 | 4 | 4 | 4 | 2 | 4 | 2 | 2 | 4 | 4 | 4 |
| 111 | 2 | 1979 | 5 | 5 | 5 | 4 | 4 | 5 | 5 | 2 | 2 | 2 | 5 | 2 | 3 | 1 | 1 | 1 | 2 | 2 | 4 | 2 | 3 | 2 | 1 | 4 | 1 | 3 |
| 117 | 2 | 1975 | 4 | 5 | 5 | 5 | 5 | 3 | 3 | 3 | 3 | 4 | 4 | 4 | 5 | 3 | 3 | 3 | 4 | 4 | 5 | 5 | 4 | 4 | 5 | 4 | 5 | 4 |
| 123 | 2 | 1974 | 4 | 5 | 5 | 5 | 5 | 5 | 5 | 5 | 4 | 4 | 5 | 2 | 5 | 5 | 3 | 3 | 2 | 4 | 5 | 4 | 2 | 4 | 2 | 5 | 5 | 5 |
| 124 | 2 | 1977 | 3 | 2 | 4 | 3 | 2 | 3 | 2 | 4 | 3 | 3 | 3 | 3 | 4 | 1 | 2 | 3 | 2 | 3 | 3 | 2 | 3 | 3 | 3 | 4 | 4 | 4 |
| 125 | 2 | 1973 | 4 | 5 | 5 | 5 | 5 | 5 | 4 | 4 | 4 | 5 | 4 | 5 | 4 | 2 | 3 | 3 | 4 | 3 | 5 | 4 | 4 | 3 | 3 | 3 | 5 | 4 |
| 126 | 2 | 1965 | 5 | 2 | 3 | 5 | 3 | 5 | 3 | 4 | 5 | 2 | 4 | 5 | 4 | 1 | 1 | 1 | 1 | 5 | 2 | 3 | 3 | 3 | 1 | 1 | 1 | 3 |
| 130 | 2 | 1973 | 5 | 4 | 5 | 4 | 5 | 4 | 4 | 4 | 4 | 3 | 4 | 5 | 3 | 2 | 2 | 2 | 2 | 2 | 4 | 2 | 2 | 2 | 3 | 4 | 3 | 4 |
| 132 | 2 | 1971 | 4 | 4 | 5 | 4 | 5 | 4 | 4 | 4 | 4 | 4 | 5 | 4 | 4 | 4 | 4 | 4 | 4 | 4 | 4 | 4 | 3 | 4 | 4 | 4 | 3 | 4 |
| 141 | 2 | 1976 | 4 | 5 | 4 | 4 | 4 | 4 | 2 | 4 | 4 | 3 | 3 | 4 | 4 | 2 | 4 | 4 | 2 | 4 | 3 | 3 | 4 | 3 | 1 | 4 | 4 | 4 |
| 151 | 2 | 1977 | 5 | 4 | 3 | 4 | 4 | 5 | 5 | 5 | 4 | 4 | 5 | 4 | 4 | 2 | 3 | 3 | 4 | 4 | 4 | 4 | 3 | 4 | 3 | 3 | 2 | 4 |
| 152 | 2 | 1976 | 4 | 4 | 3 | 4 | 5 | 4 | 4 | 4 | 4 | 4 | 4 | 4 | 3 | 3 | 2 | 4 | 3 | 2 | 4 | 4 | 3 | 1 | 2 | 5 | 3 | 4 |
| 156 | 2 | 1971 | 4 | 4 | 4 | 5 | 4 | 5 | 5 | 5 | 5 | 4 | 5 | 5 | 4 | 3 | 4 | 4 | 3 | 4 | 3 | 3 | 4 | 4 | 3 | 3 | 3 | 4 |
| 162 | 2 | 1970 | 4 | 5 | 5 | 5 | 4 | 5 | 4 | 5 | 4 | 4 | 4 | 5 | 4 | 3 | 5 | 5 | 5 | 4 | 4 | 5 | 4 | 3 | 4 | 4 | 3 | 4 |
| 167 | 2 | 1979 | 5 | 3 | 3 | 5 | 4 | 5 | 4 | 5 | 4 | 3 | 5 | 5 | 3 | 1 | 3 | 1 | 3 | 3 | 3 | 3 | 3 | 5 | 1 | 4 | 4 | 2 |
| 169 | 2 | 1970 | 1 | 1 | 4 | 3 | 5 | 1 | 1 | 1 | 3 | 1 | 1 | 1 | 4 | 2 | 1 | 2 | 3 | 3 | 1 | 1 | 3 | 1 | 1 | 4 | 5 | 1 |
| 170 | 2 | 1952 | 2 | 1 | 3 | 2 | 2 | 2 | 2 | 1 | 1 | 1 | 2 | 1 | 2 | 1 | 1 | 2 | 1 | 2 | 1 | 2 | 2 | 2 | 2 | 3 | 1 | 3 |
| 172 | 2 | 1966 | 5 | 5 | 4 | 4 | 3 | 4 | 3 | 3 | 5 | 3 | 5 | 3 | 4 | 2 | 3 | 3 | 3 | 3 | 3 | 3 | 5 | 3 | 3 | 4 | 3 | 4 |
| 175 | 2 | 1973 | 4 | 4 | 4 | 4 | 4 | 4 | 4 | 4 | 4 | 4 | 4 | 4 | 5 | 4 | 4 | 4 | 4 | 5 | 4 | 4 | 4 | 4 | 4 | 4 | 4 | 4 |
| 177 | 2 | 1977 | 2 | 3 | 3 | 3 | 2 | 2 | 1 | 2 | 2 | 2 | 3 | 2 | 3 | 3 | 4 | 3 | 4 | 4 | 2 | 3 | 2 | 3 | 3 | 3 | 3 | 3 |
| 183 | 2 | 1975 | 5 | 4 | 4 | 4 | 5 | 5 | 4 | 5 | 5 | 3 | 4 | 5 | 5 | 3 | 4 | 3 | 4 | 5 | 4 | 3 | 4 | 5 | 2 | 5 | 4 | 4 |
| 184 | 2 | 1977 | 4 | 4 | 4 | 4 | 4 | 4 | 3 | 4 | 4 | 3 | 4 | 4 | 3 | 2 | 2 | 1 | 2 | 3 | 3 | 3 | 3 | 3 | 2 | 3 | 2 | 3 |
| 187 | 2 | 1976 | 4 | 4 | 3 | 4 | 4 | 4 | 3 | 3 | 3 | 3 | 2 | 2 | 3 | 3 | 3 | 3 | 4 | 3 | 3 | 3 | 4 | 3 | 2 | 4 | 3 | 3 |
| 190 | 2 | 1979 | 4 | 4 | 5 | 4 | 3 | 4 | 3 | 3 | 3 | 3 | 3 | 2 | 2 | 2 | 1 | 2 | 3 | 3 | 3 | 2 | 1 | 2 | 2 | 2 | 2 | 1 |
| 191 | 2 | 1959 | 5 | 4 | 4 | 3 | 3 | 5 | 3 | 4 | 5 | 4 | 4 | 3 | 4 | 2 | 3 | 3 | 3 | 4 | 4 | 2 | 4 | 4 | 3 | 4 | 3 | 4 |
| 192 | 2 | 1978 | 3 | 4 | 4 | 4 | 4 | 4 | 3 | 3 | 3 | 3 | 3 | 3 | 4 | 2 | 3 | 4 | 4 | 4 | 3 | 4 | 3 | 4 | 4 | 5 | 3 | 4 |
| 195 | 2 | 1973 | 4 | 4 | 5 | 5 | 4 | 3 | 3 | 2 | 2 | 3 | 5 | 4 | 3 | 3 | 2 | 3 | 2 | 3 | 3 | 3 | 2 | 3 | 2 | 4 | 3 | 3 |
| 197 | 2 | 1978 | 5 | 5 | 5 | 5 | 5 | 5 | 3 | 5 | 4 | 2 | 5 | 4 | 5 | 3 | 4 | 4 | 4 | 3 | 2 | 1 | 4 | 4 | 1 | 5 | 4 | 4 |
| 198 | 2 | 1974 | 5 | 5 | 5 | 5 | 1 | 5 | 5 | 5 | 5 | 1 | 5 | 5 | 5 | 1 | 5 | 1 | 1 | 5 | 1 | 1 | 5 | 1 | 1 | 1 | 1 | 1 |
| 201 | 2 | 1962 | 4 | 4 | 4 | 4 | 4 | 4 | 4 | 4 | 4 | 3 | 4 | 4 | 4 | 4 | 4 | 4 | 3 | 4 | 4 | 4 | 4 | 4 | 3 | 4 | 4 | 4 |

Source: Own research.

| **Value labels** | | |
| --- | --- | --- |
|  | RESP_ID | Respondent ID |
|  | Sex | 1 woman, 2 man |
|  | Year of birth | Year of birth |
| q9_r1 | health values | 1 - not important at all, 2 - rather unimportant, 3 - hard to say, 4 - rather important, 5 – very important |
| q9_r2 | nutritional values | 1 - not important at all, 2 - rather unimportant, 3 - hard to say, 4 - rather important, 5 – very important |
| q9_r3 | shelf life | 1 - not important at all, 2 - rather unimportant, 3 - hard to say, 4 - rather important, 5 – very important |
| q9_r4 | composition of the product | 1 - not important at all, 2 - rather unimportant, 3 - hard to say, 4 - rather important, 5 – very important |
| q9_r5 | sensory properties (taste. smell) | 1 - not important at all, 2 - rather unimportant, 3 - hard to say, 4 - rather important, 5 – very important |
| q9_r6 | no preservatives | 1 - not important at all, 2 - rather unimportant, 3 - hard to say, 4 - rather important, 5 – very important |
| q9_r7 | organic/bio product | 1 - not important at all, 2 - rather unimportant, 3 - hard to say, 4 - rather important, 5 – very important |
| q9_r8 | quality certificate | 1 - not important at all, 2 - rather unimportant, 3 - hard to say, 4 - rather important, 5 – very important |
| q9_r9 | traditional recipes | 1 - not important at all, 2 - rather unimportant, 3 - hard to say, 4 - rather important, 5 – very important |
| q9_r10 | manufacturer | 1 - not important at all, 2 - rather unimportant, 3 - hard to say, 4 - rather important, 5 – very important |
| q9_r11 | country of origin of the product | 1 - not important at all, 2 - rather unimportant, 3 - hard to say, 4 - rather important, 5 – very important |
| q9_r12 | local product | 1 - not important at all, 2 - rather unimportant, 3 - hard to say, 4 - rather important, 5 – very important |
| q9_r13 | in-store availability | 1 - not important at all, 2 - rather unimportant, 3 - hard to say, 4 - rather important, 5 – very important |
| q9_r14 | on-site tastings | 1 - not important at all, 2 - rather unimportant, 3 - hard to say, 4 - rather important, 5 – very important |
| q9_r15 | loyalty programmes | 1 - not important at all, 2 - rather unimportant, 3 - hard to say, 4 - rather important, 5 – very important |
| q9_r16 | display at point of sale | 1 - not important at all, 2 - rather unimportant, 3 - hard to say, 4 - rather important, 5 – very important |
| q9_r17 | on-site sales promotions | 1 - not important at all, 2 - rather unimportant, 3 - hard to say, 4 - rather important, 5 – very important |
| q9_r18 | price | 1 - not important at all, 2 - rather unimportant, 3 - hard to say, 4 - rather important, 5 – very important |
| q9_r19 | product brand | 1 - not important at all, 2 - rather unimportant, 3 - hard to say, 4 - rather important, 5 – very important |
| q9_r20 | packaging appearance | 1 - not important at all, 2 - rather unimportant, 3 - hard to say, 4 - rather important, 5 – very important |
| q9_r21 | pack size | 1 - not important at all, 2 - rather unimportant, 3 - hard to say, 4 - rather important, 5 – very important |
| q9_r22 | income level | 1 - not important at all, 2 - rather unimportant, 3 - hard to say, 4 - rather important, 5 – very important |
| q9_r23 | product fashion | 1 - not important at all, 2 - rather unimportant, 3 - hard to say, 4 - rather important, 5 – very important |
| q9_r24 | habits | 1 - not important at all, 2 - rather unimportant, 3 - hard to say, 4 - rather important, 5 – very important |
| q9_r25 | curiosity about a new product | 1 - not important at all, 2 - rather unimportant, 3 - hard to say, 4 - rather important, 5 – very important |
| q9_r26 | preference of family members | 1 - not important at all, 2 - rather unimportant, 3 - hard to say, 4 - rather important, 5 – very important |
