# Supplementary material for: Determinants of dairy products purchase decisions among polish doctors: A gender-based analysis
Source: PLoS One. 2026 Feb 27;21(2):e0339849. doi: 10.1371/journal.pone.0339849 (PMC12948115; doi:10.1371/journal.pone.0339849)
Supplement: S2 Table — (DOCX) [file pone.0339849.s002.docx]

**Determinants of dairy products purchase decisions among polish doctors:**

**A gender-based analysis**

**Table 2. Communality of variables in exploratory factor analysis**

| **Motivators** | **Initial values** | **Values after extraction** |
| --- | --- | --- |
| health values | 1.000 | 0.648 |
| nutritional values | 1.000 | 0.610 |
| shelf life | 1.000 | 0.556 |
| composition of the product | 1.000 | 0.673 |
| sensory properties (taste, smell) | 1.000 | 0.608 |
| no preservatives | 1.000 | 0.709 |
| organic/bio product | 1.000 | 0.644 |
| quality certificate | 1.000 | 0.608 |
| traditional recipes | 1.000 | 0.614 |
| manufacturer | 1.000 | 0.647 |
| country of origin of the product | 1.000 | 0.581 |
| local product | 1.000 | 0.562 |
| in-store availability | 1.000 | 0.663 |
| on-site tastings | 1.000 | 0.623 |
| loyalty programmes | 1.000 | 0.549 |
| display at point of sale | 1.000 | 0.645 |
| on-site sales promotions | 1.000 | 0.569 |
| price | 1.000 | 0.703 |
| product brand | 1.000 | 0.737 |
| packaging appearance | 1.000 | 0.629 |
| pack size | 1.000 | 0.625 |
| income level | 1.000 | 0.557 |
| product fashion | 1.000 | 0.656 |
| habits | 1.000 | 0.681 |
| curiosity about a new product | 1.000 | 0.580 |
| preference of family members | 1.000 | 0.555 |

Source: Own study.
