# Supplementary material for: Determinants of dairy products purchase decisions among polish doctors: A gender-based analysis
Source: PLoS One. 2026 Feb 27;21(2):e0339849. doi: 10.1371/journal.pone.0339849 (PMC12948115; doi:10.1371/journal.pone.0339849)
Supplement: S3 Table — (DOCX) [file pone.0339849.s003.docx]

**Determinants of dairy products purchase decisions among polish doctors:**

**A gender-based analysis**

**Table 3. Results of exploratory factor analysis of purchase decision motives in the dairy product market (for total respondents and women and men)**

| **Motivators** | **Factors** | | | | | | | | | | | | | | |
| --- | --- | --- | --- | --- | --- | --- | --- | --- | --- | --- | --- | --- | --- | --- | --- |
|  | **1** | | | **2** | | | **3** | | | **4** | | | **5** | | |
|  | **T** | **F** | **M** | **T** | **F** | **M** | **T** | **F** | **M** | **T** | **F** | **M** | **T** | **F** | **M** |
| no preservatives | 0.819 | 0.832 | 0.816 | -0.001 | 0.011 | 0.133 | 0.091 | 0.205 | -0.001 | 0.145 | 0.052 | 0.033 | 0.094 | 0.019 | 0.123 |
| health values | 0.755 | 0.708 | 0.808 | -0.022 | 0.079 | -0.025 | 0.156 | 0.174 | -0.087 | 0.200 | 0.163 | -0.027 | 0.111 | 0.306 | 0.135 |
| organic/bio product | 0.754 | 0.739 | 0.722 | 0.259 | 0.323 | 0.072 | -0.017 | 0.040 | 0.052 | 0.042 | -0.053 | 0.116 | -0.077 | -0.047 | -0.101 |
| quality certificate | 0.724 | 0.732 | 0.713 | 0.215 | 0.148 | 0.092 | 0.066 | 0.188 | 0.261 | 0.127 | 0.176 | 0.243 | 0.126 | -0.059 | -0.034 |
| composition of the product | 0.720 | 0.764 | 0.711 | -0.151 | -0.160 | -0.006 | 0.076 | 0.234 | -0.038 | 0.222 | 0.178 | 0.069 | 0.279 | 0.054 | 0.329 |
| traditional recipes | 0.707 | 0.601 | 0.768 | 0.270 | 0.313 | 0.187 | 0.192 | -0.155 | -0.016 | 0.016 | 0.171 | 0.364 | -0.069 | 0.189 | -0.073 |
| local product | 0.693 | 0.720 | 0.658 | 0.233 | 0.220 | 0.003 | 0.079 | -0.085 | 0.211 | -0.007 | 0.119 | 0.252 | 0.147 | 0.160 | 0.061 |
| nutritional values | 0.642 | 0.691 | 0.610 | -0.071 | -0.062 | 0.127 | 0.181 | 0.195 | 0.107 | 0.137 | 0.298 | -0.152 | 0.376 | 0.151 | 0.480 |
| product fashion | 0.017 | 0.137 | -0.197 | 0.791 | 0.819 | 0.242 | 0.015 | 0.096 | 0.658 | 0.042 | 0.122 | 0.210 | 0.168 | -0.066 | -0.013 |
| on-site tastings | 0.250 | 0.262 | 0.164 | 0.720 | 0.798 | 0.470 | 0.180 | 0.064 | 0.245 | 0.062 | 0.036 | 0.367 | -0.073 | 0.063 | -0.197 |
| display at point of sale | 0.074 | 0.102 | 0.040 | 0.709 | 0.758 | 0.329 | 0.271 | 0.259 | 0.535 | 0.233 | 0.080 | 0.428 | 0.101 | 0.209 | 0.095 |
| packaging appearance | 0.018 | 0.043 | 0.061 | 0.702 | 0.679 | 0.120 | 0.116 | 0.228 | 0.787 | 0.108 | 0.237 | 0.208 | 0.333 | 0.155 | -0.020 |
| loyalty programmes | 0.137 | -0.098 | 0.381 | 0.646 | 0.707 | 0.100 | 0.102 | 0.253 | 0.275 | 0.319 | 0.228 | 0.589 | 0.000 | 0.141 | -0.066 |
| on-site sales promotions | 0.089 | 0.130 | -0.022 | 0.625 | 0.738 | 0.326 | 0.210 | 0.176 | 0.118 | 0.349 | 0.050 | 0.664 | -0.069 | 0.172 | 0.137 |
| habits | -0.009 | 0.014 | -0.029 | 0.211 | 0.234 | 0.760 | 0.792 | 0.117 | 0.109 | 0.091 | 0.103 | 0.098 | 0.019 | 0.822 | 0.121 |
| preference of family  members | 0.143 | 0.159 | 0.106 | 0.187 | 0.117 | 0.470 | 0.595 | 0.346 | 0.323 | 0.277 | 0.343 | 0.204 | 0.264 | 0.585 | 0.360 |
| sensory properties  (taste, smell) | 0.338 | 0.403 | 0.302 | 0.204 | 0.139 | 0.699 | 0.542 | 0.473 | 0.236 | 0.182 | 0.408 | 0.058 | 0.352 | 0.288 | 0.116 |
| curiosity about a new product | 0.247 | 0.408 | -0.003 | 0.485 | 0.437 | 0.631 | 0.514 | 0.175 | 0.268 | 0.114 | 0.229 | 0.424 | 0.083 | 0.361 | 0.007 |
| price | 0.095 | 0.026 | 0.207 | 0.358 | 0.341 | -0.361 | -0.097 | 0.753 | 0.313 | 0.707 | 0.198 | 0.643 | 0.240 | 0.101 | 0.148 |
| pack size | 0.189 | 0.160 | 0.260 | 0.147 | 0.276 | 0.138 | 0.297 | 0.644 | -0.186 | 0.681 | 0.234 | 0.469 | 0.128 | 0.283 | 0.509 |
| income level | 0.186 | 0.227 | 0.167 | 0.378 | 0.354 | 0.231 | 0.179 | 0.746 | 0.288 | 0.589 | -0.075 | 0.534 | 0.018 | 0.063 | 0.067 |
| in-store availability | 0.296 | 0.352 | 0.243 | 0.109 | 0.175 | 0.334 | 0.485 | 0.487 | -0.128 | 0.571 | 0.050 | 0.558 | 0.052 | 0.487 | 0.514 |
| shelf life | 0.119 | 0.248 | -0.004 | 0.013 | 0.056 | 0.104 | 0.231 | 0.423 | 0.083 | 0.351 | 0.601 | 0.081 | 0.605 | 0.089 | 0.772 |
| product brand | 0.258 | 0.216 | 0.310 | 0.465 | 0.398 | 0.506 | 0.383 | 0.035 | 0.631 | -0.113 | 0.708 | -0.058 | 0.543 | 0.183 | 0.131 |
| manufacturer | 0.402 | 0.341 | 0.466 | 0.352 | 0.243 | 0.283 | 0.314 | 0.046 | 0.570 | -0.030 | 0.652 | 0.072 | 0.512 | 0.311 | 0.103 |
| country of origin of the product | 0.483 | 0.537 | 0.442 | 0.170 | 0.205 | -0.228 | -0.130 | 0.127 | 0.411 | 0.216 | 0.467 | 0.054 | 0.505 | -0.130 | 0.476 |

Symbol designations: T-total, F- female, M - male.

Source: Own study.
